# Global profiling of CPL3-mediated alternative splicing reveals regulatory mechanisms of DGK5 in plant immunity and phosphatidic acid homeostasis

**DOI:** 10.1186/s13059-025-03529-2

**Published:** 2025-03-21

**Authors:** Sung-Il Kim, Xiyu Ma, Liang Kong, Wenbin Guo, Lahong Xu, Libo Shan, Runxuan Zhang, Ping He

**Affiliations:** 1 Department of Molecular, Cellular, and Developmental Biology, University of Michigan, Ann Arbor, MI 48109 USA; 2 Department of Biochemistry & Biophysics, Texas a&M University, College Station, TX 77843 USA; 3 Information and Computational Sciences Department, The James Hutton Institute, Dundee, UK

**Keywords:** Alternative splicing (AS), Plant immunity, CTD phosphatase, Diacylglycerol kinase, Phosphatidic acid (PA), Reactive oxygen species (ROS)

## Abstract

**Background:**

Alternative splicing of precursor mRNAs serves as a crucial mechanism to enhance gene expression plasticity for organismal adaptation. However, the precise regulation and function of alternative splicing in plant immune gene regulation remain elusive.

**Results:**

Here, by deploying in-depth transcriptome profiling with deep genome coverage coupled with differential expression, differential alternative splicing, and differential transcript usage analysis, we reveal profound and dynamic changes in alternative splicing following treatment with microbial pattern flg22 peptides in *Arabidopsis*. Our findings highlight RNA polymerase II C-terminal domain phosphatase-like 3 (CPL3) as a key regulator of alternative splicing, preferentially influencing the splicing patterns of defense genes rather than their expression levels. CPL3 mediates the production of a flg22-induced alternative splicing variant, diacylglycerol kinase 5α (DGK5α), which differs from the canonical DGK5β in its interaction with the upstream kinase BIK1 and subsequent phosphorylation, resulting in reduced flg22-triggered production of phosphatidic acid and reactive oxygen species. Furthermore, our functional analysis suggests that DGK5β, but not DGK5α, contributes to plant resistance against virulent and avirulent bacterial infections.

**Conclusions:**

These findings underscore the role of CPL3 in modulating alternative splicing dynamics of defense genes and DGK5 isoform-mediated phosphatidic acid homeostasis, shedding light on the intricate mechanisms underlying plant immune gene regulation.

**Supplementary Information:**

The online version contains supplementary material available at 10.1186/s13059-025-03529-2.

## Background

To prevent pathogen invasion, plant pattern recognition receptors (PRRs) resident on the cell surface initiate the first line of innate immune responses, termed pattern-triggered immunity (PTI), by detecting microbe-associated molecular patterns (MAMPs) [1–3]. Bacterial flagellin and its synthetic derivative, a 22-amino acid peptide known as flg22, stand as a well-studied MAMP capable of eliciting PTI responses in plants [4]. The plasma membrane (PM)-resident receptor kinase complex comprised of FLAGELLIN-SENSING 2 (FLS2) and BRI1-ASSOCIATED RECEPTOR KINASE 1 (BAK1) binds to flg22 as the immune receptor-coreceptor pair [4]. BOTRYTIS-INDUCED KINASE 1 (BIK1), a PM-associated receptor-like cytoplasmic kinase (RLCK), interacts with multiple PRRs and relays diverse downstream signaling events [5–7]. Upon receptor-coreceptor dimerization, a myriad of signaling events occurs, including the phosphorylation of BIK1-family RLCKs, the influx of Ca^2+^, generation of reactive oxygen species [8], activation of mitogen-activated protein kinases (MAPKs), and transcriptional reprogramming of defense-related genes [3, 9, 10].

MAMP perception also induces the rapid induction of phosphatidic acid (PA), a universal second messenger relaying multiple cellular signaling events [11]. Recent investigations have shown that DIACYLGLYCEROL KINASE 5 (DGK5) functions as a lipid kinase mediating MAMP-induced PA production [12–14]. The RLCKs BIK1 and RPM1-INDUCED PROTEIN KINASE (RIPK) phosphorylate DGK5 at the serine^506^ residue to enhance its activity and promote PA production upon multiple MAMP perception [12, 13]. DGK5-generated PA binds to and stabilizes the PM-resident NADPH oxidase RESPIRATORY BURST OXIDASE HOMOLOGUE D (RBOHD), an enzyme mediating the apoplastic ROS production [12, 13]. Interestingly, PRR-activated MITOGEN-ACTIVATED PROTEIN (MAP) KINASE 4 (MPK4) phosphorylates DGK5 at the threonine^446^ residue to attenuate its activity [13]. In addition, DGK5 plays a role in intracellular immune receptor nucleotide-binding site leucine-rich repeat protein (NLR)-mediated immunity with a similar phospho-regulation [13]. Thus, the opposing phosphorylation of DGK5 by BIK1/RIPK and MPK4 regulates PA homeostasis and ROS production in plant immunity.

Different MAMPs trigger profound and largely overlapping gene transcriptional reprogramming in plants [15, 16]. Gene transcription is largely regulated by the phosphorylation status of RNA POLYMERASE II (RNAPII) CARBOXY (C)-TERMINAL DOMAIN (CTD) [17–19]. CTD consists of various conserved heptapeptide repeats with the consensus sequence of Y_1_S_2_P_3_T_4_S_5_P_6_S_7_, and its phosphorylation is dynamically regulated by various CTD kinases and phosphatases during transcription [17, 18]. Interestingly, MAMP perceptions induce the rapid and transient phosphorylation of *Arabidopsis* CTD, mediated by cyclin-dependent kinases CDKCs and CTD PHOSPHATASE-LIKE 3 (CPL3) [20]. CPL3 is a homolog of yeast TFIIF-ASSOCIATING CTD PHOSPHATASE (FCP1) [21], which specifically dephosphorylates CTD Ser2 residue [20]. CTD Ser2 phosphorylation mainly regulates transcription elongation and mRNA processing, especially recruiting splicing factors for mRNA splicing [22–24].

mRNA splicing serves as a critical cellular process essential for maintaining the integrity of the transcriptome in eukaryotic cells [22, 24]. Furthermore, alternative splicing (AS) significantly broadens the diversity of mRNA transcripts and proteins that can arise from a single gene, thereby enabling increased complexity and regulation in gene expression [24, 25]. Emerging evidence indicates the role of AS in orchestrating the expression of immune receptor and signaling genes, thereby optimizing immune responses and coordinating cellular defense mechanisms [26]. Notably, AS plays a crucial role in modulating the expression and function of several intracellular Toll-IL-1 receptor homology region [27]-type NLR immune receptors, including tobacco *N* gene, *Arabidopsis RESISTANCE TO PSEUDOMONAS SYRINGAE 4* (*RPS4*) and *SUPPRESSOR OF npr1-1, CONSTITUTIVE 1* (*SNC1*) [28–30]. Additionally, AS impacts the regulation of genes encoding cell surface-resident receptor-like kinases (RLKs) *SNC4* and *CHITIN ELICITOR RECEPTOR KINASE 1* (*CERK1*) [31]. Another example of AS-mediated regulation involves the mRNA of *CALCIUM-DEPENDENT PROTEIN KINASE 28* (*CPK28*), a negative regulator of PTI, which undergoes AS upon activation by the phytocytokine Plant Elicitor Peptide PEP1. This process results in the production of an intron-retained transcript encoding a truncated protein with decreased kinase activity [32]. Moreover, pathogens have evolved strategies to target host spliceosome components, enabling them to reprogram mRNA splicing and thus dampen plant immunity [33, 34].

In addition to its pivotal role in plant immunity, AS has been shown to modulate responses to abiotic stresses in various species. For example, rice splicing factor OsSCR106 plays a crucial role in AS under stress conditions such as salt and cold. Loss-of-function mutations in *OsSCR106* resulted in hypersensitivity to these stresses, highlighting its importance in stress adaptation through proper pre-mRNA splicing [35]. Similarly, several *Arabidopsis* splicing factors have been shown to modulate cold responses, with mutations in these genes leading to hypersensitivity to low temperatures and impaired growth [36]. Furthermore, AS in *Dendrobium catenatum* orchids contributes to cold stress regulation by generating specific splice variants of stress-related genes to enhance survival under freezing conditions [37]. Together, these studies illustrate that AS is a versatile regulatory mechanism across species, allowing plants to fine-tune gene expression in response to both biotic and abiotic stresses.

The complexity of the AS landscape, compounded by the dynamic nature of host–pathogen interactions and the heterogeneity of cellular responses, presents inherent difficulties in capturing the breadth of splicing events. Traditional RNA sequencing (RNA-seq), typically generating 5–20 million (M) reads per sample, is limited by its sensitivity to detect low-abundance isoforms, biases related to transcript length, and the ability to distinguish between isoforms with subtle sequence variations [38]. In this study, we conducted an extensive RNA-seq analysis, with 120 M reads per sample, on *Arabidopsis* wild-type (WT) Col-0 and *clp3-3* mutants following flg22 elicitation, coupled with the newly developed 3D RNA-seq pipeline for the analysis of differentially expressed genes (DEG), differential alternative splicing (DAS), and differential transcript usage (DTU) [39]. We obtained a comprehensive landscape of AS events triggered by the flg22 treatment. Notably, CPL3 emerged as a key regulator of flg22-induced AS events, rather than influencing overall gene transcriptional changes. Moreover, *DGK5α*, a splicing variant of *DGK5* that encodes a truncated protein lacking the C-terminal calmodulin-binding motif, is produced abundantly and further induced upon flg22 treatment in a CPL3-dependent manner, compared to full-length *DGK5* transcript, *DGK5β*. Unlike DGK5β, DGK5α is unable to be phosphorylated by BIK1 with impaired lipid kinase activities, thereby compromising its function in plant PRR- and NLR-mediated immunity. Our findings suggest that the upregulation of this non-functional variant upon MAMP perception may contribute to the attenuation of the PA burst, serving as a transcriptional feedback regulation.

## Results

### In-depth global profiling of alternative splicing events in response to flg22 treatment

**Fig. 1:**
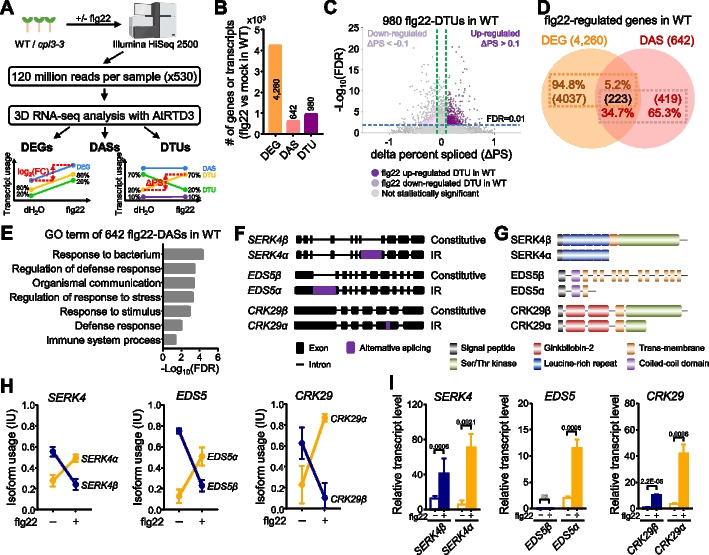
Global profiling of alternative splicing events in response to flg22 treatment. **A** Scheme of in-depth transcriptome profiling for alternative splicing in response to flg22. Two-week-old plate-grown wild-type (WT) Col-0 and *cpl3-3* mutant seedlings treated with H_2_O (mock) or 100 nM flg22 for 60 min were subjected to RNA isolation and sequencing. To capture the transcripts with low abundance, RNA-seq was performed with Illumina HiSeq 2500 to obtain 120 million reads per sample with the paired-end 150-bp read length, which is about 530 × coverage of *Arabidopsis* transcriptome. The sequenced reads were aligned to the AtRTD3 reference transcriptome for quantification. The 3D RNA-seq analysis was performed to identify differentially expressed genes (DEGs), differentially alternatively spliced genes (DASs), and transcripts with differential transcript usage (DTUs). The bottom left panel shows an example of DEGs with two differentially expressed transcripts, where changes in abundance between conditions (dH_2_O and flg22 treatments) are measured by log_2_ fold change. Total gene expression is represented in the blue line, which is the sum of the expression of all individual transcripts (green and yellow lines). The percentage values denote the expression ratios of individual transcripts relative to the total gene expression. The bottom right panel illustrates examples of DAS and DTU. For a DAS gene, it must have more than one transcript, and changes in expression between individual transcripts (green, yellow, and purple lines) and the total gene expression (blue line) are compared between conditions. The change in percent spliced (ΔPS) is the percentage change in the abundance of a transcript relative to the total gene expression. For a gene to be classified as DAS, at least one transcript has a |ΔPS|≥ 0.1. In DTU analysis, individual transcripts show different expression patterns compared to other transcripts of the same gene. DTU is identified by comparing the change in expression of each transcript to the average expression change of the other transcripts within the same gene. In this example, the transcripts represented with green and yellow lines, but not with the purple line, are DTUs. **B** Flg22 treatment triggers transcriptional changes in gene expression and alternative splicing in WT plants. The Y-axis indicates the numbers of flg22-triggered DEGs and DAS genes, and DTU transcripts. The flg22-triggered up-/downregulated DEGs were identified based on an absolute value of fold change (|FC|) ≥ 2 and false discovery rate (FDR) < 0.01 between mock and flg22 treatment. The flg22-triggered DASs and flg22-triggered DTUs were selected based on an absolute delta percent spliced (|ΔPS|) ≥ 0.1 and FDR < 0.01 between mock and flg22 treatment. **C** Volcano plot of flg22-triggered DTUs in WT. Up- and downregulated DTUs in response to flg22 in WT were depicted by a volcano plot. Purple and pale purple dots represent up- and downregulated flg22-DTUs, respectively. The DTUs with non-statistically significant differences were indicated as gray. The Y-axis denotes − log_10_(FDR), while the X-axis shows ΔPS values. The cut-off lines for FDR = 0.01 and ΔPS =  ± 0.1 were indicated as blue and green dashed lines, respectively. **D** Limited overlapping between flg22-DEGs and flg22-DASs. The Venn diagram between flg22-DEGs (orange circle) and flg22-DASs (pink circle) in WT plants shows the percentages and corresponding gene numbers indicated in each group. **E** Gene ontology (GO) analysis of flg22-DASs in WT. The statistically enriched gene ontology terms were identified based on the frequency of up-/downregulated flg22-DASs annotated to their frequency in the genome with the cut-off of fold enrichment ≥ 1 and false discovery rate (FDR) < 0.05. **F** Diagrams of gene structures for three representative flg22-DASs in WT. Solid lines indicate introns, black boxes represent exons, and purple boxes denote alternatively spliced regions. Constitutive, constitutive splicing isoform; IR, intron retention. **G** Diagrams of protein domains for three representative flg22-DASs in WT. Proteins encoded by constitutive and splicing variant transcripts are designated as β and α forms, respectively. Distinct functional domains with various colored boxes were labeled in the figure. **H** Relative isoform abundances of three representative flg22-DASs in WT. The isoform usage (IU) was calculated by the percentage abundance of a transcript compared to the total expression of the gene. The blue line represents constitutive splicing transcript (β form), which was defined by a major isoform containing all exons among all splicing variants, and the yellow line represents alternative splicing transcripts (*α* form), respectively. The expression levels of individual transcripts were retrieved from RNA-seq data. **I** RT-qPCR analysis of individual splicing variants from three representative flg22-DAS genes. Two-week-old seedlings were treated with or without 100 nM flg22 for 60 min for RT-qPCR analysis with primers specific to each splicing variant. Relative expressions of target transcripts were normalized to *UBQ10*, and data are shown with mean ± S.D. from three biological repeats (*n* = 3). Data were analyzed by unpaired two-tailed Student’s *t*-test between mock- and flg22-treatment. Non-statistically (ns) and statistically significant differences with the corresponding *p* values were indicated in the figure

Capturing the full gene-splicing events, particularly for those occurring with low abundance, is critical to reveal the heterogeneity of various gene isoforms from different AS events. To achieve this, we substantially increased the sequencing depth of RNA-seq with an average of 120 M 150-bp long paired-end sequencing reads per sample, which is about 530 × coverage of the AtRTD3 reference transcriptome [40] (Fig. 1A; Additional file 1: Table S1). We first analyzed the RNA-seq datasets from the *Arabidopsis* ecotype Col-0 seedlings treated with 100 nM flg22 or H_2_O (mock) for 60 min, which is commonly used to induce PTI in *Arabidopsis* [20, 41, 42]. No significant batch-to-batch variation from three biological repeats was found using principal component analysis (PCA) with RNA-seq data from total transcripts and genes (Additional file 2: Fig. S1A). The flg22 treatment was validated by well-studied early PTI marker genes, including *WRKY30*, *WRKY33*, and *FLG22-INDUCED RECEPTOR-LIKE KINASE1* (*FRK1*) from the RNA-seq data (Additional file 2: Fig. S1B). The trimmed and normalized datasets were subjected to the 3D RNA-seq analysis to identify DE genes, DAS genes, and DTU transcripts (Fig. 1A). The pipeline of 3D RNA-seq analysis incorporates state-of-the-art bioinformatic methods and normalizes the expression with data-driven optimal parameters to improve the analytical accuracy [39]. Out of 40,932 genes from the AtRTD3 reference transcriptome [40], 4260 genes (10.4%), comprising 2741 upregulated and 1519 downregulated genes, were identified as flg22-regulated differentially expressed genes (flg22-DEGs) based on cut-off of |fold change|≥ 2 and false discovery rate [FDR] < 0.01 compared to the mock treatment (Fig. 1B; Additional file 2: Fig. S1C; Additional file 1: Table S2).

To determine splicing variant changes in response to flg22 treatment, we analyzed transcript isoform usage (IU) by calculating the percentage abundance of each transcript relative to the total expression of each gene [40]. Subsequently, we determined the values of delta percentage spliced (ΔPS), which represents the difference in IUs between mock and flg22 treatment. If an IU of any transcript from a gene is significantly changed by flg22 treatment (|ΔPS|≥ 0.1 and FDR < 0.01), we called the gene and transcript DAS and DTU, respectively (Fig. 1A). A total of 642 flg22-regulated DAS genes (flg22-DASs), accounting for 1.6% of the total 40,932 genes from AtRTD3, were identified in WT (Fig. 1B; Additional file 1: Table S3). Their corresponding 980 flg22-regulated DTU transcripts (flg22-DTUs) comprised 527 upregulated and 453 downregulated transcripts (Fig. 1C; Additional file 1: Table S4). Notably, 223 genes were shared between flg22-DEGs and flg22-DASs, representing 5.2% of flg22-DEGs and 34.7% of flg22-DASs (Fig. 1D). This observation suggests that flg22 predominantly regulates distinct sets of genes exhibiting either differential expression or splicing to optimize plant defense mechanisms. The functional consequences of AS are reflected in the changes in isoform ratios [43]. We examined isoform switching (IS) events, where a pair of transcripts reverse their relative abundance between two different conditions, representing one of the most prominent isoform usage changes [39]. Our analysis revealed that 77 flg22-DAS genes, accounting for 12%, exhibited ISs between two flg22-DTU transcripts (Additional file 2: Fig. S1D). Gene ontology (GO) enrichment analysis revealed that among 642 flg22-DASs in WT, there was significant enrichment of terms related to immunity, such as response to the bacterium, regulation of defense response, and immune system process (Fig. 1E; Additional file 1: Table S5). Taken together, our findings demonstrate that flg22 elicitation induces significant alterations in both gene expression and AS patterns. Importantly, each of these processes largely regulates distinct groups of genes, highlighting the complexity and specificity of the plant immune response to flg22.

Given that different transcript isoforms of a gene can encode the same functional protein sequence, the flg22-DTU transcripts were annotated for their gene structure and translated functional domains. Among flg22-DTUs, the transcripts having constitutive splicing events from the majority of individual splicing junctions were defined as the constitutive isoform [44], which was denoted as the beta (β) form here based on *DGK5* AS nomenclature (see below). The transcripts that encode different protein sequences from the constitutive ones were denoted as the alpha (α) form. Most, if not all, flg22-DASs only have β and α forms. Occasionally, the transcripts encoding protein sequences different from β and α forms were denoted as the gamma (γ) form. Several well-known immunity-related genes, including *SOMATIC EMBRYOGENESIS RECEPTOR-LIKE KINASE 4* (*SERK4*), *ENHANCED DISEASE SUSCEPTIBILITY 5* (*EDS5*), and *CYSTEINE-RICH PROTEIN KINASE 29* (*CRK29*), were identified among flg22-DASs, with substantial read counts (Fig. 1F and G; Additional file 2: Fig. S1E). The *SERK4α* has an intron retention (IR) splicing variant at the 7th intron (Fig. 1F), resulting in a premature SERK4α protein after the leucine-rich repeat (LRR) domain (Fig. 1G). Lacking the kinase domain, SERK4α is unlikely to be functional. An IR splicing variant at the first intron of *EDS5* leads to a truncated and non-functional EDS5α protein that lacks domains after the first transmembrane domain (Fig. 1F and G). The *CRK29α* bears an IR at the last intron, leading to a truncated protein with an incomplete kinase domain (Fig. 1F and G). Interestingly, *SERK4*, *EDS5*, and *CRK29* display IS between two isoforms at 60 min upon flg22 treatment (Fig. 1H). The ratio of their α form transcripts increased, whereas the ratio of the β form transcripts decreased upon PTI activation (Fig. 1H). The production of nonfunctional α form transcripts might be a mechanism for plants to balance the immune response via counteracting the functional β forms. The transcript levels of each isoform obtained from RNA-seq data were confirmed by RT-qPCR with primers specific to each isoform (Fig. 1I; Additional file 2: Fig. S1F and G). Notably, the induction of both α and β transcript isoforms for *SERK4*, *EDS5*, and *CRK29* peaked at 60 min, and then quickly declined upon flg22 treatment (Additional file 2: Fig. S1G).

### CPL3 exerts a profound role in regulating alternative splicing dynamics upon flg22 elicitation

CPL3 negatively regulates plant immunity by modulating RNAPII CTD Ser2 dephosphorylation in response to flg22 treatment [20]. Given that RNAPII CTD Ser2 phosphorylation is essential in recruiting splicing components [45], we investigated the role of CPL3 in flg22-regulated AS with the *cpl3-3* mutant compared to WT plants for in-depth 3D RNA-seq analysis (Fig. 1A). The *cpl3-3* (SALK_094720) has a T-DNA insertion in the 6th exon of AT2G33540 (*CPL3*), resulting in a truncation of its protein sequence within the FCP homology domain [20] (Additional file 2: Fig. S2A). The mutant was confirmed by PCR genotyping analysis (Additional file 2: Fig. S2B). The growth phenotype of the mutant, observed from 2 to 4 weeks of age, showed no significant differences compared to WT Col-0 plants (Additional file 2: Fig. S2C). The RNA-seq samples of mock- and flg22-treated WT and *cpl3-3* seedlings were examined, revealing no significant batch-to-batch variation using the PCA test (Additional file 2: Fig. S1A). Notably, only 1.1% (441 out of 40,932) genes were basal DEGs, and 0.1% (40 out of 40,932) were basal DAS genes between WT and *cpl3-3* without flg22 treatment (Additional file 1: Tables S6 and S7), suggesting that CPL3 does not regulate basal transcriptional and AS alterations, consistent with our previous report [20].

**Fig. 2:**
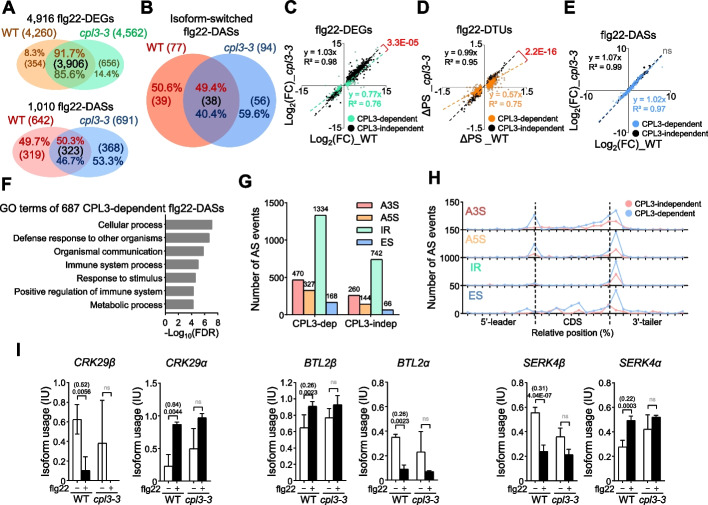
CPL3 profoundly affects flg22-triggered alternative splicing events. **A** CPL3 plays a more important role in flg22-triggered DASs than flg22-triggered DEGs. Venn diagram shows the percentage and gene numbers between up-/downregulated flg22-DEGs (top) and flg22-DASs (bottom) in WT and *cpl3-3*. The flg22-DEGs were identified based on (|FC|) ≥ 2 and FDR < 0.01. The flg22-DASs were selected based on |ΔPS|≥ 0.1 and FDR < 0.01. **B** CPL3 regulates isoform-switched DASs. The isoform-switched DASs were identified when a pair of transcripts reversed their relative abundance between mock and flg22 treatments. A Venn comparison plot illustrates the overlap of isoform-switched DASs between WT and *cpl3-3* in response to flg22 treatment. **C**–**E** Correlation analyses of flg22-DEGs, flg22-DTUs, and flg22-DASs between WT and *cpl3-3*. The gene expression changes or proportional isoform usages from CPL3-dependent flg22-DEGs (**C**), flg22-DTUs (**D**), and flg22-DASs (**E**) were represented as green, orange, and blue dots with trend lines, respectively. The gene expression changes or proportional isoform usages from CPL3-independent flg22-DEGs, -DTUs, and -DASs were represented as black dots with trend lines. The log_2_(FC) values were used for flg22-DEGs and flg22-DASs, and the ΔPS values were used for flg22-DTUs. The X-axis and Y-axis values are from WT or *cpl3-3*, respectively. The trend line equation and the Pearson correlation coefficiencies (*R*^2^) between WT and *cpl3-3* were labeled. The correlations were analyzed by the Chow test between CPL3-dependent and -independent flg22-DEGs, flg22-DTUs, and flg22-DASs. Non-statistically (ns) and statistically significant differences with *p* values were indicated in the figure. **F** GO analysis using 687 of CPL3-dependent flg22-DASs. The statistically enriched gene ontology terms were identified based on the frequency of CPL3-dependent flg22-DASs annotated to their frequency in the genome with the cut-off of fold enrichment ≥ 1 and false discovery rate (FDR) < 0.05. **G** The total number of flg22-triggered AS events in CPL3-dependent (CPL3-dep) and CPL3-independent (CPL3-indep) flg22-DTUs. The different types of AS events, namely alternative 3′ splicing (A3S), alternative 5′ splicing (A5S), intron retention (IR), and exon skipping (ES), were depicted as red, orange, green, and blue bars, respectively. **H** The distribution of flg22-triggered AS events in CPL3-dependent and CPL3-independent flg22-DTUs. The number of flg22-triggered AS events across 5′-leader, CDS, and 3′-tailer is indicated by red and blue lines for WT and *cpl3-3*, respectively. The relative position was calculated as the average of the alternative coordinates of the AS event, scaled by the length of the CDS, and then converted to a percentage based on their full lengths, dividing into 10% windows. The position of the ATG start codon and stop codon are indicated by black dashed lines, separating the 5′-leader and 3′-tailer from the CDS. **I** Relative isoform abundances of three representative CPL3-dependent flg22-DASs in WT and *cpl3-3*. The isoform usage (IU) was calculated by the percentage abundance of a transcript compared to the total transcripts of the gene. The *β* and *α* represent constitutive and alternative splicing isoforms, respectively. The absolute ΔPS values (in parentheses) and FDR values between mock (white bar) and flg22 treatment (black bar) are indicated at the top of each comparison. Non-statistically significant comparisons (ns) were indicated as gray. The expression levels of individual transcripts were retrieved from RNA-seq data

In the *cpl3-3* mutant, we identified 4562 flg22-DEGs, comprising 2785 upregulated and 1777 downregulated genes, and 691 flg22-DASs with 526 upregulated and 447 downregulated flg22-DTU transcripts (Additional file 1: Tables S2, S3, and S4). Out of the 4562 flg22-DEGs in *cpl3-3*, 3906 genes (85.6%) overlapped with flg22-DEGs in WT. Similarly, 91.7% of flg22-DEGs in WT overlapped with those in *cpl3-3*, suggesting that CPL3 only regulates a small portion of flg22-DEGs. Importantly, only about 50% of flg22-DASs overlapped between WT and *cpl3-3*, which leaves another 50% of flg22-DASs specific to WT or *cpl3-3* (Fig. 2A). Similarly, about 50% of IS flg22-DASs were shared between WT and *cpl3-3* (Fig. 2B). This analysis indicates that CPL3 plays a more profound role in flg22-regulated AS than its regulation in gene expression.

Heatmap analysis of flg22-DEGs in WT and *cpl3-3* classified flg22-DEGs into six groups: UP_WT (genes induced in WT, but not in *cpl3*); UP_common (genes induced in both WT and *cpl3*); UP_*cpl3* (genes induced in *cpl3*, but not in WT); DN_WT (genes repressed in WT, but not in *cpl3*); DN_common (genes repressed in both WT and *cpl3*); DN_*cpl3* (genes repressed in *cpl3*, but not in WT) (Additional file 2: Fig. S3A). Among the total of 4916 flg22-DEGs in WT and/or *cpl3-3*, 1010 flg22-DEGs specific to WT or *cpl3-3* were designated as CPL3-dependent flg22-DEGs (Additional file 1: Table S8; Additional file 2: Fig. S3B), while the remaining as CPL3*-*independent flg22-DEGs. The Pearson correlation of expression changes between WT and *cpl3-3* for CPL3-dependent flg22-DEGs was lower than that for CPL3-independent flg22-DEGs (*R*^2^ values of 0.76 and 0.98, respectively) (Fig. 2C). Moreover, a slope of gene expression change trend-line for CPL3-dependent flg22-DEGs was significantly reduced towards *cpl3-3* compared to CPL3-independent flg22-DEGs (*p* value from the Chow test was 3.3E − 05; Fig. 2C). These results suggest that CPL3 suppresses the expression of upregulated CPL3-dependent flg22-DEGs while enhancing the expression of downregulated CPL3-dependent flg22-DEGs, consistent with our previous results [20]. Apparently, CPL3 has a more profound role in flg22-DEGs than basal DEGs.

Heatmap analysis of 1497 flg22-DTUs in WT and/or *cpl3-3* classified flg22-DTUs into six groups: UP_WT (induced IU in WT, but not in *cpl3*); UP_common (induced IU in both WT and *cpl3*); UP_*cpl3* (induced IU in *cpl3*, but not in WT); DN_WT (repressed IU in WT, but not in *cpl3*); DN_common (repressed IU in both WT and *cpl3*); DN_*cpl3* (repressed IU in *cpl3*, but not in WT) (Additional file 2: Fig. S3C). Among them, 1041 flg22-DTUs specific to WT or *cpl3-3* were designated as CPL3-dependent flg22-DTUs, while the remaining as CPL3-independent flg22-DTUs (Additional file 1: Table S9; Additional file 2: Fig. S3D). Similar to flg22-DEGs, the difference in IU of CPL3-dependent flg22-DTUs exhibited a lower correlation than that of CPL3-independent flg22-DTUs (*R*^2^ values of 0.75 and 0.95, respectively) (Fig. 2D). Consistently, the slope of the IU difference (ΔPS) trend-line for CPL3-dependent flg22-DTUs showed a significant reduction towards *cpl3-3* (*p* value from the Chow test was 2.2E − 16; Fig. 2D), supporting that CPL3 suppresses IU of upregulated CPL3-dependent flg22-DTUs while enhancing downregulated CPL3-dependent flg22-DTUs. The log_2_(FC) values of CPL3-dependent flg22-DASs were compared to CPL3*-*independent flg22-DASs to determine whether the IU changes of CPL3*-*dependent flg22-DASs are due to their gene expression changes. The Pearson correlation coefficients of CPL3*-*dependent and -independent flg22-DASs from WT and *cpl3-3* were similar (0.97 vs. 0.99; Additional file 1: Table S10; Fig. 2E), suggesting that CPL3 regulates flg22-triggered AS by modulating specific IU rather than controlling gene expression. GO enrichment analysis using 687 of CPL3-dependent flg22-DASs indicates that immune-related terms, such as defense response to other organisms, immune system process, and positive regulation of immune system pathway, were significantly enriched compared to the distribution of GO terms across all genes in the genome (Fig. 2F; Additional file 1: Table S5). Taken together, these results indicate that CPL3 exerts a pronounced effect on flg22-triggered AS events largely independent of its regulation on gene expression level.

Among different types of AS events, alternative 3′ splicing (A3S), alternative 5′ splicing (A5S), intron retention (IR), and exon skipping (ES) were identified as the four major types in flg22-triggered DTUs (Fig. 2G), consistent with a previous report [46]. The total numbers of all four events were substantially increased in CPL3-dependent DTUs compared to CPL3-independent DTUs (Fig. 2G). We further plotted the relative positions of these AS events in flg22-triggered DTUs on a scaled gene structure, divided into 10% windows, consisting of the 5′-leader, coding sequence (CDS), and 3′-tailer. CPL3 has an effect on all four AS events. Apparently, it has a more pronounced role from the end of the CDS to the beginning of the 3′-tailer (Fig. 2H), consistent with its function in dephosphorylating RNAPII CTD Ser2, which is enriched during transcriptional elongation to termination [22–24]. Additionally, a quantitative comparison of isoform usage changes between WT and *cpl3-3* indicates that among the four AS events, IR showed significantly higher ΔPS values in *cpl3-3* than in WT at the CDS and 3′-tailer (Additional file 2: Fig. S3E). These findings suggest that while CPL3 regulates all four major flg22-triggered AS events, it plays a particularly significant role in IR, both in terms of the number of AS events and the ΔPS values at the CDS and 3′-tailer.

We also analyzed in detail a few CPL3-dependent flg22-DASs with previously known functions in plant immunity for their gene structures and protein domains from corresponding DTU transcripts. Interestingly, several membrane-resident proteins related to PTI exhibited CPL3-dependent IU changes upon flg22 treatment. Those include genes encoding RLKs, such as *SERK4*, *CRK29*, *FRK1*, *BAK1-INTERACTING RECEPTOR-LIKE KINASE 1* (*BIR1*), and *BACK TO LIFE 2* (*BTL2*), and a gene encoding calcium transporter *AUTO-INHIBITED CA*^*2*+^ *ATPASE 12* (*ACA12*). CPL3 either regulated the IU of both α and β forms of transcripts (*SERK4*, *BTL2*, *CRK29*, *FRK1*, and *ACA12*), or only α form of transcripts (*BIR1*) (Fig. 2I; Additional file 2: Fig. S4A–C). Two *MAP KINASES*, *MPK3* and *MPK12*, were also among CPL3-dependent flg22-DASs (Additional file 2: Fig. S4A–C). Interestingly, unlike most *RLK* genes, the constitutive β forms of *MPK3* and *MPK12* transcripts were induced, whereas their α forms were suppressed upon flg22 treatment (Additional file 2: Fig. S4A).

Since CPL3 plays a crucial role in regulating AS events, we investigated whether CPL3 colocalized with spliceosome components, specifically ARGININE/SERINE-RICH ZINC KNUCKLE-CONTAINING PROTEIN 33 (RSZ33) and SMALL NUCLEAR RIBONUCLEOPROTEIN U1 SUBUNIT 70 (U1-70 k) within the nucleus [47]. RSZ33 and U1-70 K have been widely used as representative markers for spliceosome components, with distinct roles in the mRNA splicing process and their relevance to AS [48, 49]. CPL3-GFP was co-expressed with either RSZ33-RFP or U1-70 k-RFP in *Arabidopsis* protoplasts and then treated with or without flg22 to examine their colocalization. Notably, the two spliceosome components, RSZ33 and U1-70 k, displayed speckles that did not overlap with the diffused CPL3 signals in the nucleoplasm with and without flg22 treatment (Additional file 2: Fig. S3F). This observation suggests that CPL3 may not directly interact with the spliceosome in regulating AS, but rather operates through the regulation of CTD Ser2 phosphorylation. Consistently, the C-terminal region of CPL3 (CPL3C), which contains the catalytic FCP homology domain [20], dephosphorylated the flg22-triggered CTD Ser2 phosphorylation (Additional file 2: Fig. S3G).

CPL3 regulates the transcription and alternative splicing of *DGK5.*

**Fig. 3:**
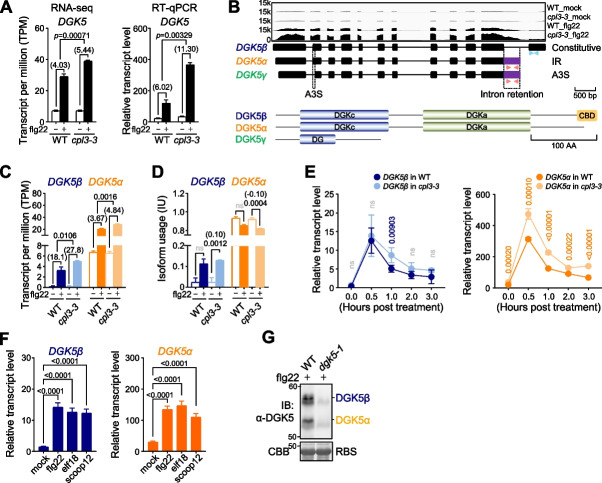
CPL3 negatively regulates flg22-induced *DGK5* gene expression and alternative splicing. **A** Flg22-induced *DGK5* expression is elevated in *cpl3-3*. The gene expression levels of *DGK5* were quantified with transcript per million (TPM) values from RNA-seq data (left) and independently confirmed by RT-qPCR (right). Two-week-old seedlings were treated with dH_2_O or 100 nM flg22 for 60 min for RT-qPCR analysis. Gene expressions of *DGK5* were normalized to *UBQ10*, and data are shown with mean ± S.D. from three biological repeats (*n* = 3). Data were analyzed by unpaired two-tailed Student’s *t*-test between WT and *cpl3-3*. The *p* values are indicated at the top of the figure. **B** Diagrams of gene structures and protein domain annotations for three *DGK5* isoforms. The read coverage for the *DGK5* gene from each sample was visualized using an Integrative Genomics Viewer (IGV) (top). Introns are represented by solid lines, while exons and alternatively spliced regions are depicted by black and purple boxes, respectively (middle). Two pairs of primers distinguishing *DGK5β* and *DGK5α* isoforms from the last exon are indicated by blue and red arrows, respectively. While the primers amplifying *DGK5β* could also amplify *DGK5γ*, the expression levels of *DGK5γ* were negligible with less than 1 TPM in the RNA-seq analysis. The intron retention (IR) site is indicated by the dashed lines on the top of the panel. The alternative 3′ splice site (A3S) is indicated by the dashed lines on the bottom of the panel. The constitutive protein denoted as DGK5β (blue) contains the DGK catalytic domain (DGKc), DGK accessory domain (DGKa), and calmodulin-binding domain (CBD) (bottom). DGK5α (orange) lacks the CBD, and DGK5γ only contains partial DGKc. **C** The flg22-induced upregulation of *DGK5β* and *DGK5α* is elevated in *cpl3-3*. Transcript levels of *DGK5β* and *DGK5α* were quantified with TPM values from RNA-seq data. The mock and flg22 treatments are depicted as opened and closed bars, respectively. The fold changes were analyzed by comparing TPM values between mock and flg22 treatments and indicated at the top of each comparison in parentheses. Data were analyzed by unpaired two-tailed Student’s *t*-test between WT and *cpl3-3*, and the *p* values are indicated at the top of each comparison. **D** The IUs of *DGK5β* and *DGK5α* in WT and *cpl3-3* upon flg22 treatment. The IU was calculated by the percentage abundance of each transcript compared to the total transcripts of the gene. The absolute ΔPS values (in parentheses) and FDR values between mock (opened bar) and flg22 treatment (closed bar) are indicated at the top of each comparison. Data were analyzed by unpaired two-tailed Student’s *t*-test between WT and *cpl3-3*, and the *p* values or non-statistically significant differences (ns) are indicated at the top of each comparison. **E** Flg22-triggered transient induction of *DGK5β* and *DGK5α* in WT and *cpl3-3*. Two-week-old seedlings were treated with 100 nM flg22 for the indicated times for RT-qPCR analysis. Transcript expressions of *DGK5β* and *DGK5α* were normalized to *UBQ10*, and data are shown with mean ± S.D. from three biological repeats (*n* = 3). Data were analyzed by unpaired two-tailed Student’s *t*-test between WT and *cpl3-3* mutant. The *p* values between WT and *cpl3-3* are indicated at the top of each comparison. Non-statistically differences comparisons (ns) are indicated as gray. **F** Multiple elicitors induce the transcript levels of *DGK5β* and *DGK5α*. Two-week-old seedlings were treated with mock, 100 nM flg22, elf18, or scoop12 for 60 min for RT-qPCR analysis. Data are shown as mean ± S.D. from four biological repeats (*n* = 4) analyzed by one-way ANOVA followed by Tukey’s test with the *p* values indicating statistical difference. **G** The α-DGK5 antibody detects endogenous DGK5β and DGK5α proteins. Total proteins were extracted from 2-week-old WT and *dgk5-1* seedlings, followed by immunoblotting using an α-DGK5 antibody with Rubisco (RBS) stained by Coomassie brilliant blue (CBB) as a loading control. The polyclonal α-DGK5 antibody was generated by using full-length DGK5 proteins as an antigen from rabbits

The PA biosynthesis gene *DGK5*, recently implicated in plant immunity [12–14], also exhibited CPL3-dependent AS events. While *DGK5* displayed a significant upregulation upon flg22 treatment in both WT and *cpl3-3*, the flg22-induced *DGK5* expression was notably higher in *cpl3-3* than in WT, suggesting that CPL3 exerts a negative regulatory role on *DGK5* expression (Fig. 3A).

Interestingly, forty-two transcript isoforms of *DGK5* were identified from AtRTD3 and were categorized into three protein groups based on their translation and functional domain annotation by FGENESH (www.softberry.com). The full-length protein sequence, referred to as DGK5β, contains DGK catalytic domain (DGKc), DGK accessory domain (DGKa), and calmodulin-binding domain (CBD) [12, 13, 50]. The truncated isoform lacking the CBD was named DGK5α, similar to other DGK homologs in plants [50, 51], and another truncated isoform containing only partial DGKc was DGK5γ (Additional file 2: Fig. S5A). The *DGK5α* isoform arises from the intron retention at the last intron, resulting in CBD truncation at the carboxyl terminus (Fig. 3B). The *DGK5γ* isoform underwent alternative splicing at the 3′ end of the first intron, leading to the alternative 3′ splice site (A3S) and resulting in a truncated protein at the DGKc domain (Fig. 3B). The aggregated TPM values from RNA-seq data were utilized to examine the total transcript levels for each isoform. The expression levels of *DGK5γ* were negligible, with less than 1 TPM in both mock and flg22 treatment conditions (Additional file 2: Fig. S5B). Conversely, expression levels of *DGK5β* and *DGK5α* were significantly induced by flg22 treatment (Fig. 3C). Moreover, the flg22-induced expression of *DGK5β* and *DGK5α* was further enhanced in *cpl3-3* compared to WT, suggesting that CPL3 negatively regulates the expression of both *DGK5β* and *DGK5α* (Fig. 3C). Additionally, we assessed the IUs of two *DGK5* isoforms by examining the proportion of their transcripts relative to the total *DGK5* transcripts (Additional file 2: Fig. S5C). While the IUs of *DGK5β* and *DGK5α* were not significantly altered in WT upon flg22 treatment, their IUs were significantly and oppositely changed in *cpl3-3*, further supporting the regulatory role of CPL3 in *DGK5* AS events (Fig. 3D).

RT-qPCR analysis with primers specific to each isoform (Fig. 3B) confirmed the flg22-triggered induction of both *DGK5β* and *DGK5α*. This induction was transient, peaking at half an hour post-treatment (hpt) and gradually returning to basal levels by 3 hpt (Fig. 3E). Notably, CPL3 regulated *DGK5α* levels across all time points following flg22 treatment (Fig. 3E). Additionally, the expression level of *DGK5α* was about 10 times higher than *DGK5β*, both with and without flg22 treatment. In addition to flg22, the MAMP elf18 and the phytocytokine scoop12 also significantly induced the expression of both *DGK5β* and *DGK5α* (Fig. 3F). Different *DGK5* AS isoforms are also induced by abiotic stresses. For example, salt stress induced the expression of both *DGK5β* and *DGK5α*, whereas cold stress led to an increase of only *DGK5α* isoform (Additional file 2: Fig. S5D and E). However, these inductions were not influenced by CPL3 (Additional file 2: Fig. S5D and E). This finding suggests that CPL3-mediated *DGK5* AS is specific to certain stimuli, such as MAMP treatments.

We also generated DGK5 antibodies using full-length DGK5β proteins as an antigen to detect endogenous DGK5β and DGK5α proteins. To distinguish the subtle 2 kDa size difference between DGK5β (57.4 kDa) and DGK5α (55.3 kDa) proteins, total proteins from flg22-treated WT and *dgk5-1* leaves were separated using the Protein II XL SDS-PAGE system and then immunoblotted with an α-DGK5 antibody. The migration distances of the proteins from the top of the gel to the developed bands were measured and compared to a standard curve generated using protein markers (Additional file 2: Fig. S5F). The DGK5 antibody successfully detected both endogenous DGK5β and DGK5α with the expected band sizes in WT but not in *dgk5-1* (Fig. 3G). Together, these data indicate that *DGK5* transcribes as two major isoforms, *DGK5β* and *DGK5α*, both of which are induced by multiple MAMPs, partially dependent on CPL3.

### DGK5α has compromised diacylglycerol kinase activity and cannot be phosphorylated by BIK1

**Fig. 4:**
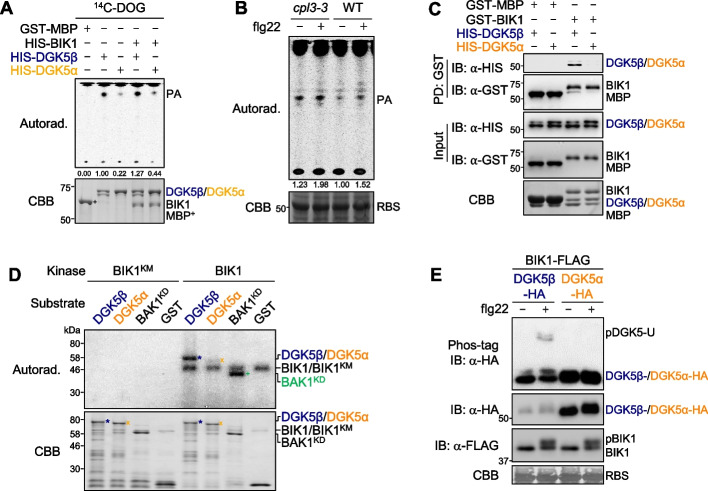
DGK5α has compromised diacylglycerol kinase activity and cannot be phosphorylated by BIK1. **A** DGK5α exhibits a compromised diacylglycerol kinase activity. The recombinant GST-MBP, HIS-DGK5β, or HIS-DGK5α proteins were incubated with [^14^C]-DOG in a reaction buffer containing ATP for 30 min. Chloroform-soluble products were separated by the thin-layer chromatography (TLC) plate, and PA was detected by autoradiography (Autorad., top). The amount of PA levels is quantified based on the band intensities using ImageJ software. The PA level produced by DGK5β (second lane) was set as 1, and relative PA levels were labeled on the bottom of the autoradiography panel. The protein loading is shown by CBB staining on the bottom. **B** The *cpl3-3* mutant displays the elevated PA production. Cell lysates from WT and *cpl3-3* seedlings with/without 100 nM flg22 treatment for 10 min were incubated with [^14^C]-DOG in a reaction buffer for 60 min. Total lipids were separated by the TLC plate placed in an acidic solvent system, and PA was detected by autoradiography (top panel). The protein loading is shown by CBB staining on the bottom. The band intensities corresponding to PA were quantified using the ImageJ software. The PA level without flg22 treatment in WT was set as 1, and relative PA levels were labeled on the bottom of the autoradiography panel. **C** BIK1 interacts with DGK5β, but not DGK5α, in an in vitro pull-down assay. Recombinant GST-BIK1 proteins and GST-MBP proteins (control) were immobilized on glutathione sepharose beads and incubated with HIS-DGK5β or HIS-DGK5α proteins for the pull-down assay. Eluted proteins were subjected to immunoblotting with an α-HIS or α-GST antibody (PD: GST; top two panels), and proteins before the pull-down assay are shown as input (middle two panels). The total proteins are stained by CBB (bottom panel). **D** BIK1 phosphorylates DGK5β, but not DGK5α in vitro. The in vitro kinase assay was performed using purified GST-BIK1 or GST-BIK1^KM^ as a kinase and GST, GST-BAK1^KD^, GST-DGK5β, or GST-DGK5α as the substrates using [γ-^32^P]-ATP. BIK1^KM^ is a BIK1 kinase-dead mutant, and BAK1^KD^ is the BAK1 truncation with the kinase domain alone, which does not carry auto-phosphorylation activity. Phosphorylation was analyzed by autoradiography (Autorad.) (top), and protein loading is shown by CBB staining (bottom). **E** DGK5β, but not DGK5α, shows the upper shifted band upon flg22 treatment in the presence of BIK1. DGK5β-HA or DGK5α-HA was co-expressed with BIK1-FLAG in protoplasts from WT plants, followed by 100 nM flg22 treatment for 10 min. Total proteins were separated with Mn.^2+^-Phos-tag (top panel) or regular SDS-PAGE (middle two panels), followed by immunoblotting with an α-HA or α-FLAG antibody. Rubisco stained by CBB from the regular SDS-PAGE serves as a loading control (bottom panel)

DGK5 functions as a diacylglycerol kinase, catalyzing the phosphorylation of diacylglycerol (DAG) to produce PA [13]. To investigate whether DGK5α, lacking the CBD, retains diacylglycerol kinase activity, we conducted an in vitro radio-labeled thin-layer chromatography (TLC) assay. Recombinant DGK5β or DGK5α were incubated with their substrate, ^14^C-labeled unsaturated DAG analog, 1,2-dioleoyl-sn-glycerol (DOG), and the lipids were separated on thin-layer chromatography. Consistent with the previous report [13], DGK5β could phosphorylate DOG into PA, whereas DGK5α showed a substantially reduced PA production compared to DGK5β (Fig. 4A). The DGK5α activity was still low even in the presence of HIS-BIK1 proteins (Fig. 4A). Additionally, we compared the endogenous DGK activities using cell lysates from WT and *cpl3-3*. Consistent with the increased transcripts of *DGK5* in *cpl3-3*, both basal and flg22-induced PA levels were elevated in *cpl3-3* compared to WT plants (Fig. 4B).

RLCKs, including BIK1 and RIPK, directly interact with DGK5β and phosphorylate DGK5β at Ser^506^ in response to MAMP signals [12, 13]. Ser^506^ is located within the CBD of DGK5, which is absent in DGK5α. Consistently, recombinant six histidine-tagged DGK5β, but not DGK5α, directly pulled down glutathione S-transferase (GST)-tagged BIK1 in an in vitro pull-down assay (Fig. 4C). Furthermore, GST-BIK1, but not its kinase-dead mutant GST-BIK1^KM^, phosphorylated GST-DGK5β, but not GST-DGK5α, in an in vitro kinase assay (Fig. 4D). As a positive control for the kinase assay, GST-BIK1 phosphorylated the kinase domain of BAK1 (GST-BAK1^KD^) [52]. BIK1 phosphorylates DGK5β in vivo, resulting in an upper mobility shift band (pDGK5-U) in Phos-tag SDS-PAGE when they were co-expressed in protoplasts upon flg22 treatment (Fig. 4E) [13]. Importantly, the pDGK5-U band was not detected for DGK5α upon flg22 treatment (Fig. 4E). Together, the results indicate that DGK5α, lacking the CBD, exhibits compromised diacylglycerol kinase activities and does not interact with BIK1, nor can it be phosphorylated by BIK1.

### DGK5α is deficient in mediating PTI and ETI

**Fig. 5:**
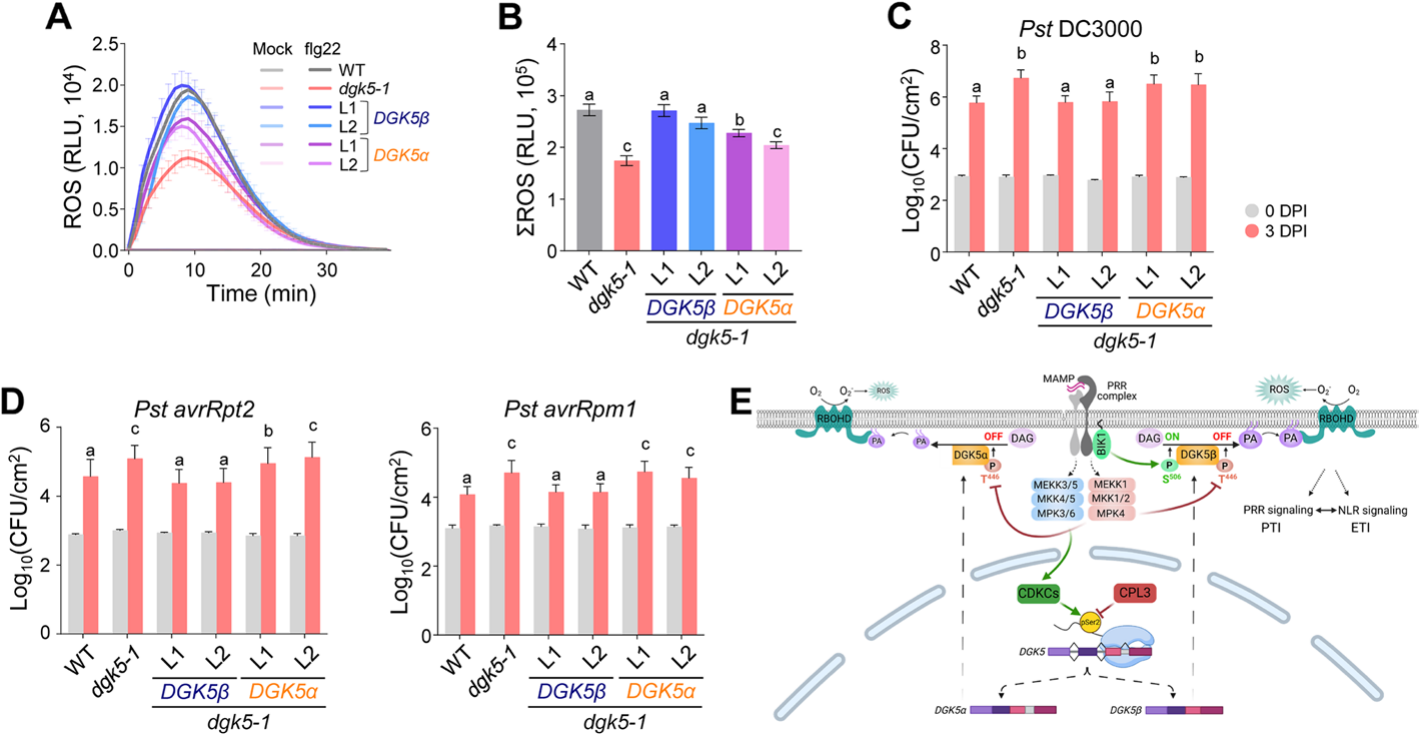
DGK5α is defective in plant PTI and ETI [48]. DGK5β, but not DGK5α, restores flg22-induced ROS burst in the *dgk5-1* mutant. Leaf disks from 4-week-old soil-grown WT, *dgk5-1*, and two independent transgenic lines carrying *p35S*::*DGK5β-HA* or *p35S*::*DGK5α-HA* in the *dgk5-1* background were treated with or without 100 nM flg22, and the ROS production was measured as relative light units (RLU) by a luminometer for the indicated time (**A**). Total ROS levels between 0 and 30 min (ΣROS) are shown as mean ± S.E.M. (*n* = 24, biologically independent samples) analyzed by one-way ANOVA followed by Tukey’s test (**B**). Different letters (a, b, and c) indicate significant differences (*p* value < 0.05). **C** DGK5β, but not DGK5α, complements the *dgk5-1* mutant defect of plant disease resistance against virulent bacterial pathogen *Pst* DC3000. Leaves from 4-week-old soil-grown WT, *dgk5-1*, *p35S::DGK5β-HA/dgk5-1*, or *p35S::DGK5α-HA/dgk5-1* transgenic plants were hand-inoculated with *Pst* DC3000 bacterial suspension at 5 × 10^5^ cfu/ml. Bacterial growth was measured at 0 and 3 days post-inoculation (dpi). Data are shown as mean ± S.D. (*n* = 6, biologically independent samples) analyzed by one-way ANOVA followed by Tukey’s test for multiple comparisons. Different letters (a and b) indicate significant differences (*p* value < 0.05). **D** DGK5β, but not DGK5α, complements the *dgk5-1* mutant defect of plant disease resistance against avirulent bacterial pathogens *Pst* DC3000 carrying *avrRpt2* or *avrRpm1*. The experiments were performed similarly as in **C** using the bacterial suspension of *Pst* DC3000 *avrRpt2* or *Pst* DC3000 *avrRpm1* at 5 × 10^5^ cfu/ml. Data are shown as mean ± S.D. (*n* = 6, biologically independent samples) analyzed by one-way ANOVA followed by Tukey’s test for multiple comparisons. Different letters (a, b, and c) indicate significant differences (*p* value < 0.05). **E** A model of CPL3-mediated *DGK5* AS in regulating PA homeostasis and plant immunity. MAMP perception by the PRR complex triggers BIK1 phosphorylation and activation of two MAPK cascades. BIK1 phosphorylates DGK5 at Ser^506^ and enhances its activity for PA production. In contrast, PRR-activated MPK4 phosphorylates DGK5 at Thr^446^, leading to reduced DGK5 activity and PA production. In addition, MAMP perception induces RNAPII CTD phosphorylation through MPK3/6-activated CDKCs, counteracted by the CTD phosphatase CPL3. The CDKC-CPL3-mediated CTD phosphorylation regulates *DGK5* AS, producing two isoforms, *DGK5β* and *DGK5α*. DGK5β, which can be phosphorylated by BIK1, positively regulates DGK5 activity and plant immunity. In contrast, DGK5α, unable to be phosphorylated by BIK1, is nonfunctional and likely negatively regulates PA production through MPK4-mediated Thr^446^ phosphorylation to maintain PA homeostasis. Further, PA binds and stabilizes RBOHD in mediating ROS production in plant PRR-mediated PTI and NLR-mediated ETI. The figure was created with BioRender

The *dgk5* mutants exhibit compromised MAMP-triggered ROS production [12, 13]. We sought to delineate the contribution of different *DGK5* isoforms to PTI responses by introducing *DGK5β-HA* or *DGK5α-HA* constructs under the *35S* promoter into the *dgk5-1* mutant. Two independent transgenic lines for each isoform were selected for further analysis. Consistent with previous reports, *DGK5β* fully restored the defect of *dgk5-1* in flg22-induced ROS production [13] (Fig. 5A and B). However, *DGK5α* either did not complement the *dgk5-1* defect in flg22-induced ROS production or provided only partial restoration (Fig. 5A and B). Moreover, the *dgk5* mutants exhibited increased susceptibility to infection by the virulent *Pseudomonas syringae* pv. *tomato* (*Pst*) DC3000 [13]. While *DGK5β* fully rescued the defect of *dgk5-1* to *Pst* DC3000 infections, *DGK5α* failed to provide complementation (Fig. 5C).

Furthermore, the *dgk5* mutants are compromised in effector-triggered immunity [14] triggered by *Pst* DC3000 carrying *avrRpt2* or *avrRpm1* [13]. Similar to responses against virulent *Pst* DC3000, *DGK5β* fully restored the *dgk5-1* defect to infections with *Pst* DC3000 *avrRpt2* or *avrRpm1*, whereas *DGK5α* did not complement this defect (Fig. 5D). In summary, these findings highlight the defective role of DGK5α in plant PTI and immunity against virulent and avirulent bacterial pathogens.

## Discussion

mRNA splicing is an important post-transcriptional regulatory mechanism governing gene expression in both plant and metazoan innate immunity [26, 53]. Despite recognizing that several plant immune-related genes undergo AS, our understanding of the global landscape of AS events following immune elicitation remains limited. Here, we performed an in-depth RNA-seq analysis and revealed the AS landscape of *Arabidopsis* immune response following MAMP flg22 treatment. We further show that RNAPII CTD phosphatase CPL3 plays an essential role in regulating the flg22-triggered AS landscape, likely through the dephosphorylation of CTD Ser2 residue. Moreover, using DGK5 as a model, which undergoes CPL3-dependent AS, we demonstrate the significance of different DGK5 AS isoforms in maintaining PA homeostasis and modulating plant immunity (Fig. 5E).

Traditional transcriptome analyses, such as regular RNA-seq and microarrays, are limited in accurately detecting AS events, particularly those involving low-abundance isoforms and complex splice junctions. In contrast, long-read RNA-seq, such as PacBio and Oxford Nanopore, offer a solution by generating reads spanning entire transcripts, thus providing a more comprehensive view of AS events [54–57]. While long-read RNA-seq has advantages in observing splice junctions, detecting low-abundance isoforms, and characterizing complex AS events, it comes with drawbacks like higher error rates, lower throughput, and increased sequencing costs compared to short-read platforms. To balance these trade-offs, we performed RNA-seq analysis with 120 M 150-bp paired-end sequencing reads per sample. This approach, which provides extensive coverage of the *Arabidopsis* transcriptome (~ 530 × coverage), ensures accurate detection of low abundance reads and complex isoform diversity while maintaining sequencing accuracy, throughput, and compatibility with traditional computational tools. Notably, the recently released *Arabidopsis* reference transcriptome AtRTD3, generated using high-resolution single-molecule long-read sequencing, offers advantages over other datasets, such as TAIR, by providing a more comprehensive and accurate representation of the *Arabidopsis* transcriptome, especially in capturing rare or complex isoforms [40]. This comprehensive approach not only identified well-expressed genes like *SERK4* and *CRK29*, as reported previously [46], but also uncovered low-expressed genes such as *DGK5* and *EDS5*, as flg22-DASs, highlighting the robustness and sensitivity of our strategy in detecting AS events across a wide range of expression levels.

In this study, we identified 4260 flg22-DEGs and 642 flg22-DASs in WT, indicating that gene expression changes are more pronounced than AS events upon immune elicitation. Notably, only a small portion of flg22-DEGs and flg22-DASs overlap. This pattern is consistent with observations in plant responses to abiotic stresses [36, 58]. For instance, while 7302 genes were identified as cold-DEGs, 2442 genes were identified as cold-DASs, with a substantial portion of these gene groups being distinct and non-overlapping [36]. These findings support the hypothesis of independent evolution in the regulation of transcript levels and AS, suggesting that stress response regulation involves two separate mechanisms: changes in gene expression levels and AS, each governing a different gene set [59]. The minimal overlap between genes regulated at the expression and AS levels upon flg22 treatment mirrors the largely non-overlapping MAMP-induced transcriptional and translational landscapes [60]. These observations suggest the complexity of immune responses, which coordinate distinct sets of genes regulated at transcriptional, post-transcriptional, and translational levels to achieve a robust and balanced immune response.

The CTD phosphatase CPL3 specifically modulates RNAPII CTD Ser2 dephosphorylation in transcriptional regulation [20]. Phosphorylation of CTD Ser2 is crucial for the recruitment of spliceosome components, such as U2 small nuclear ribonucleoprotein particle (snRNP) and U2 snRNP auxiliary factor 65 (U2AF65) [45]. Two conserved splicing factors, SUPPRESSOR OF ABI3-5 (SUA) and REQUIRED FOR SNC4-1D 2 (RSN2), which associate with U2 snRNP, are involved in regulating the AS of RLK genes *SNC4* and *CERK1* [31]. We found that genes encoding spliceosome components, such as *SR30*, *RS31*, *RS31A*, and *SR34*, were differentially spliced upon flg22 treatment (Additional file 1: Table S3). However, the roles of these spliceosome components in plant immunity remain unclear. Notably, our data indicate that CPL3 does not co-localize with the spliceosome components RSZ33 and U1-70k in the nucleus.

AS can be regulated by either transcriptional speed [the cis-regulatory kinetic model] or spliceosome activity [the trans-regulatory recruitment model] [61]. Previous studies have implicated spliceosome components, such as MOS4, MOS12, LSM4, and SR45, in the regulation of AS in defense genes during plant immune responses [28, 62–64]. Given that CPL3 does not directly interact with spliceosome components, it is plausible that CPL3 indirectly regulates AS of defense genes upon immune elicitation through modulation of RNAPII CTD Ser2 phosphorylation as CTD Ser2 phosphorylation could serve as a marker for recruiting splicing machinery to target intron sites [8, 45, 65]. Alternatively, CPL3 could influence transcriptional speed through the modulation of transcription elongation kinetics mediated by CTD Ser2 phosphorylation rather than by recruiting spliceosome components. Additionally, MPK4 has been proposed to play an important role in flg22-DASs as it regulates the AS of genes encoding several splicing factors and immune-related protein kinases [46]. MPK3 and MPK6 also directly phosphorylate CTD kinases CDKCs, thereby modulating CTD phosphorylation upon flg22 perception [20]. It would be interesting to determine whether MPK4 also regulates AS through the modulation of CTD phosphorylation dynamics.

Two splicing isoforms of *DGK5*, designed as *DGK5α* and *DGK5β*, were previously identified [50, 51]. In our current study, we further elucidated the functional differences between these isoforms. Specifically, we found that DGK5α, lacking the CBD, exhibits reduced DGK activity and PA production compared to DGK5β. This observation was consistent with our findings that DGK5α failed to restore flg22-induced ROS production and resistance to both virulent and avirulent pathogens in the *dgk5-1* mutant background, supporting the crucial role of PA production in plant immunity. The necessity of the CBD for DGK5 interaction with BIK1 and subsequent BIK1-mediated phosphorylation further highlights the importance of BIK1 in regulating DGK5 activities. Our findings are corroborated by a recent report showing the compromised functional activities of DGK5α in PA biosynthesis, ROS production, and plant immunity [12]. Notably, among seven DGKs in *Arabidopsis*, only DGK5 contains the CBD [66], suggesting a potential involvement of Ca^2+^ signaling in the regulation of DGK5 activities. Additionally, we demonstrated that the *DGK5* transcripts were negatively regulated by CPL3. Consistently, the *cpl3* mutants exhibited enhanced PA levels compared to WT, supporting our previous finding that the *cpl3* mutants displayed enhanced resistance to bacterial and fungal infections [20].

## Conclusions

As PA serves as a crucial second messenger, maintaining its homeostasis is essential for normal growth and defense. In our recent research, we demonstrated that MAMP-activated MPK4 phosphorylates DGK5 at Thr^446^ to attenuate DGK5 activity and PA production, resulting in a transient PA burst [13]. We show here that upon MAMP perception, plants produce a high amount of truncated *DGK5α* transcripts encoding non-functional proteins. This might represent another mechanism by which plants maintain PA homeostasis through the regulation of *DGK5* AS events (Fig. 5E). It is also fully possible that these AS forms may have other unexplored biological functions, whether directly involved in plant immunity or in other cellular processes.

## Methods

### Plant materials and growth conditions

The *Arabidopsis thaliana* Columbia-0 (Col-0) ecotype was used as WT, and the T-DNA insertion knockout mutants *cpl3-3* (SALK_094720), *dgk5-1* (SAIL_1212_E10), and *p35S::DGK5β-HA/dgk5-1* were reported previously [13, 20]. The *p35S::DGK5α-HA/dgk5-1* transgenic plants were generated using *pCAMBIA1300-p35S::DGK5α-HA* binary construct by floral dipping method. The *cpl3-3* mutant (SALK_094720) was genotyped by PCR with primers listed in Additional file 1: Table S11.

All *Arabidopsis* plants were grown in soil (Metro Mix 366, Sunshine LP5 or Sunshine LC1, Jolly Gardener C/20 or C/Gs, USA) in a growth chamber at 20–23 °C, 50% relative humidity, and 75–100 μE m^−2^ s^−1^ light with a 12-h light/12-h dark photoperiod for 4 weeks before pathogen infection assay, protoplast isolation, and ROS assay. For RNA expression analysis such as RNA-seq and RT-qPCR assays, seeds were sterilized, stratified for 2 days at 4 °C in the dark, and germinated on vertical half-strength Murashige and Skoog (½MS) medium plates containing 1% (w/v) sucrose, 0.5% agar and 2.5 mM MES at pH 5.8, and grown under the same condition as above for 2 weeks. The seedlings were transferred to ½MS liquid medium for another day before treatment with different chemicals or elicitors.

### Sample preparation and RNA isolation for RT-qPCR and RNA-sequencing

For RT-qPCR, 2-week-old seedlings grown on vertical ½MS plates were transferred into a 24-well plate containing 500 μl liquid ½MS medium for 1 day, and then treated mock or 100 nM elicitors (elf18, flg22, and scoop12) for 1 h. Two seedlings were placed in a single well, and a total of at least six seedlings were used for each treatment. For salt stress treatment, 3-week-old soil-grown plants were watered with 100 mM of NaCl for 4 h. For cold stress treatment, 3-week-old soil-grown plants were incubated at 4 °C for 3 days. Three leaves from different plants were collected in one biological replicate.

Total RNA was isolated from the above samples using TRIzol reagent (Invitrogen, USA). One microgram of total RNA was treated with RNase-free DNase I (NEB, USA), and then was reverse transcribed to synthesize the first-strand cDNA with M-MuLV reverse transcriptase (NEB, USA) and oligo (dT) 18-mer primer.

For RNA-sequencing, 2-week-old seedlings of Col-0 and the *cpl3-3* mutant germinated on ½ MS agar plates were treated with 100 nM flg22 or mock for 1 h. The total RNA was extracted by TRIzol, quantified using a Qubit (Thermo Fisher Scientific, USA), and qualities were assessed using a Bioanalyzer (Agilent Technologies, USA). The messenger RNA was enriched from 1 μg of total RNA using a QIAseq FastSelect–rRNA Plant Kit (Qiagen, USA). The RNA-seq libraries were prepared by reverse transcription, end-repairing, and adaptor-ligation using an NEBNext Ultra II RNA Library Prep Kit for Illumina (NEB, USA). Samples from three independent biological repeats were prepared for paired-end RNA-sequencing using Illumina HiSeq 2500 platform with a 150 bp of read length at the Texas A&M Institute for Genome Sciences and Society (College Station, TX, USA). An average of 120 million reads were obtained for each sample.

### Data processing and transcript quantification

RNA-seq reads with low sequencing quality or reads with sequencing adaptors were filtered by Trim_Galore version 0.6.5 [a wrapper of the Cutadapt program [67]]. The quality of the clean reads was evaluated using FastQC version 0.11.9. After passing quality control, the expression of transcripts was quantified against the AtRTD3 [40] using Salmon version 0.14.0 [68]. The transcript quantifications were imported into the 3D RNA-seq application for expression data pre-processing, and differential expression and alternative splicing analysis [39]. Read counts were generated by using the Tximport R package with LengthScaledTPM method [69]. Aligned read counts from three biological replicates were merged and visualized for read coverages using Integrative Genomics Viewer (IGV) [27]. Transcripts were deemed as expressed if they had 1 count per million read (CPM) in at least two samples. Expressed genes were those with at least one expressed transcript. The low-expressed genes and transcripts were filtered before proceeding with downstream analysis. Gene and transcript expression were normalized across samples with a trimmed mean of M value for fair comparisons between the conditions [70].

### Identification of DEG, DAS, and DTU, and gene ontology analysis

The basal-DEGs were determined by employing an adjusted Benjamini-Hochberg (BH) *t*-test *p* value < 0.01 and an absolute log_2_(FC) ≥ 1 between WT and *cpl3-3* without flg22 treatment. Similarly, basal differential alternative splicing (basal-DAS) events were identified by comparing isoform usage between WT and *cpl3-3* samples without flg22 treatment. DAS genes were selected based on a BH-adjusted *p* value < 0.01 and an absolute value of ΔPS (defined as the difference in isoform usage between samples) ≥ 0.1 between WT and *cpl3-3* samples without flg22 treatment. The flg22-DEG, -DAS, and -DTU analyses were focused on WT_flg22 versus WT_mock and *cpl3*_flg22 versus *cpl3*_mock. Within each comparison, we identified DEGs based on the following criteria: adjusted BH *t*-test *p* value < 0.01 and absolute log_2_(FC) ≥ 1 between mock and flg22 treatments. The flg22-DAS genes were identified by comparing the log_2_(FC) of each transcript associated with a gene to the log_2_(FC) for the entire gene between mock and flg22 treatments (i.e., if any of the transcripts show as a DTU, that gene was defined as DAS). The flg22-DTU transcripts were examined by comparing the log_2_(FC) of each gene transcript isoform against the weighted average log_2_(FC) of all remaining transcript isoforms. DTU transcripts were selected based on BH-adjusted *p* value < 0.01 and the absolute value of ΔPS ≥ 0.1 between mock and flg22 treatments.

The IDs of DAS genes were uploaded to the functional gene annotation website PANTHER 18.0 [71] for gene ontology enrichment (GO) analysis. Enriched GO terms related to biological processes were considered significant when a BH-adjusted *p* value is below 0.05. SUPPA v2.3 was used to generate the AS events and their corresponding ΔPSs from the DAS genes [72]. The relative position of AS events was calculated as the average of the two alternative coordinates of that AS event scaled by the length of CDS.

### RT-qPCR analysis

The quantitative RT-PCR (RT-qPCR) was performed using iTaq SYBR green Supermix (Bio-Rad, USA) with primers targeting β or α form of *CRK29*, *DGK5*, *SERK4*, and *EDS5* listed in Additional file 1: Table S11 in a Bio-Rad CFX384 Real-Time PCR System (Bio-Rad, USA). The expression of indicated genes was normalized to *UBQ10*. The data analysis was performed using a two-sided Student’s *t*-test or one-way ANOVA followed by Tukey’s test for multiple comparisons. The *DGK5β* and *DGK5α* specific primers were designed to target exclusive sequences from 11th intron and its intron retention regions for *DGK5β* and *DGK5α*, respectively.

### Plasmid construction and transgenic plant generation

The plant gene expression vector *pHBT*, under the control of the CaMV *35S* promoter, was employed for protoplast assays, with BIK1, CPL3C, and DGK5β tagged with HA, as previously detailed [13]. The cDNA of *DGK5α* was amplified from Col-0 cDNA using primers with BamHI at the 5′-terminus and StuI at the 3′-terminus. Subsequently, the amplified product was digested with BamHI and StuI and ligated into the *pHBT* vector, incorporating the HA epitope tag at the C-terminus, utilizing the ClonExpress II One-Step Cloning Kit (Vazyme, China) following manufacturer protocols. For recombinant protein isolation, the *DGK5α* coding sequences (CDSs) within the *pHBT* vector were sub-cloned into *pET28a* with BamHI and StuI digestion. The constructs of BAK1^KD^, BIK1, BIK1^KM^, and DGK5β, fused with GST or HIS tag, for recombinant proteins from *Escherichia coli* were reported previously [13, 73].

Binary vectors were constructed by sub-cloning *DGK5α* into the binary vector *pCAMBIA1300* through BamHI/StuI digestion, yielding *pCAMBIA1300-p35S::DGK5α-HA* constructs. Transgenic plants were generated using *Agrobacterium tumefaciens*-mediated floral dipping. Screening of transgenic plants was conducted using hygromycin (50 μg/ml) and confirmation of transgene expression was achieved through immunoblotting (IB) using an α-HA antibody.

### Protoplast isolation and DGK5 mobility shift assays

Protoplast isolation and gene expression assays followed a previously established method with minor modifications [74]. Protoplasts were transfected with pairs of constructs (including the empty vector as a control, 100 μg DNA for 500 μl protoplasts at a density of 2 × 10^5^/ml for each sample) and incubated at 25 °C for 12 h. After treatment with 100 nM flg22 for 10 min, protoplasts were collected, lysed, and subjected to immunoblotting (IB) analysis. Total proteins were separated in 8% SDS-PAGE gels containing 20 μM Phos-tag™ (FUJIFILM Wako Chemicals, Japan) and 200 mM MnCl_2_. Immunoblotting with α-HA-HRP (1:2000, Roche, USA) or α-FLAG-HRP antibodies (1:2000, Sigma-Aldrich, USA) was performed.

### *Recombinant protein isolation and *in vitro* kinase assays*

Fusion proteins from the *E. coli* BL21 strain were induced in LB medium (1% tryptone, 0.5% yeast extracts, 1% NaCl) supplemented with 0.25 mM isopropyl β-D-1-thiogalactopyranoside (IPTG) at 16 °C for 12–18 h. Glutathione S-transferase (GST) fusion proteins were purified with Pierce glutathione agarose (Thermo Scientific, USA), and HIS fusion proteins were purified with Pierce Ni–NTA agarose beads (Thermo Scientific, USA) according to the manufacturer protocols.

The in vitro kinase assays were carried out with 0.5 μg of indicated kinase proteins and 5 μg of substrate proteins in the 30 μl kinase reaction buffer (10 mM Tris–HCl, pH 7.5, 5 mM MgCl_2_, 2.5 mM EDTA, 50 mM NaCl, 0.5 mM DTT, 50 μM ATP, and 1 μCi [γ-^32^P]-ATP). After shaking at speed 60 rpm for 2 h at 24 °C, the reactions were stopped by adding 4 × SDS loading buffer, and proteins were separated by 10% SDS-PAGE. Phosphorylated proteins were analyzed by autoradiography.

### Protein pull-down assays and antibody production

Recombinant GST-MBP or GST-BIK1 proteins were incubated with 10 μl pre-washed glutathione agarose beads in 300 μl incubation buffer (20 mM Tris–HCl, pH 7.5, 100 mM NaCl, 0.1 mM EDTA, and 0.2% Triton X-100) at 4 °C for 30 min in a rotator. Immobilized protein beads were washed twice with washing buffer (20 mM Tris–HCl, pH7.5, 300 mM NaCl, 0.1 mM EDTA, and 0.1% Triton X-100), followed by incubation with 20 μg bovine serum albumin (BSA, Sigma-Aldrich, USA) in 300 μl incubation buffer at 4 °C for 30 min. Protein beads were then washed twice with washing buffer, and then incubated with 2 μg HIS-DGK5β or HIS-DGK5α proteins in 300 μl incubation buffer at 4 °C for another 1 h in a mini shaker at a speed of 60 rpm. Protein beads were collected and washed three to four times with the washing buffer. Proteins were analyzed by immunoblotting with indicated antibodies.

The HIS-DGK5β proteins were used for α-DGK5 antibody production in rabbits (Abmart, China). The concentration of 0.5 μg/ml of α-DGK5 antibody was used for immunoblotting (IB) analysis to examine endogenous DGK5β and DGK5α levels. The molecular weight of DGK5β and DGK5α are determined based on their migration distance on SDS-PAGE according to [75].

### Pathogen infection assays

*Pseudomonas syringae* pv. *tomato* (*Pst*) DC3000, *Pst* DC3000 carrying the effector *avrRpt2* (*Pst* a*vrRpt2*) or *avrRpm1* (*Pst* a*vrRpm1*) was cultured overnight at 28 °C in King’s B medium (10 g protease peptone, 0.75 g K_2_HPO_4_, 10 ml 50% glycerol for 500 ml) supplemented with 2 mM MgSO_4_ and appropriate antibiotics (50 μg/ml rifamycin or kanamycin). Leaves from 4-week-old plants were hand-inoculated with bacterial suspension using a needleless syringe. To measure in planta bacterial growth, two leaf disks were collected as a set, punched, and ground in 100 μl ddH_2_O. A total of six sets were sampled. Serial dilutions were plated on TSA medium (1% tryptone, 1% sucrose, 0.1% glutamic acid, and 1.5% agar) containing 25 μg/ml rifamycin or kanamycin. Plates were incubated at 28 °C, and bacterial colony-forming units (cfu) were counted at 0 and 3 days post-incubation.

### Subcellular localization assay

Protoplasts from 3.5-week-old *Arabidopsi*s leaves were transfected with a pair of constructs of *pHBT-CPL3-GFP* and *pmAEV-U1-70 k-RFP* or *pmAEV-RSZ33-RFP* (10 μg DNA for 50 μl protoplasts at a density of 2 × 10^4^/ml for each sample) and incubated at 25 °C for 12 h. The fluorescence signals of GFP and RFP fusion proteins were observed using a Leica TCS SP8 confocal laser scanning microscope (Leica, Germany). The excitation wavelength of GFP and RFP is 488 nm and 588 nm, respectively. The emission wavelength for detecting GFP and RFP is 490–530 and 590–620 nm, respectively.

### Detection of ROS burst

ROS measurement was performed using a luminol-based approach as previously described with minor modification [13]. In brief, the third or fourth pair of true leaves from 4-week-old soil-grown *Arabidopsi*s plants were punched into leaf disks using a cork borer (5 mm in diameter). Leaf disks were incubated in 150 μl ddH_2_O in a 96-well plate overnight with gentle shaking on a rocker with a 12-h light/12-h dark photoperiod. Water was replaced with 100 μl reaction solution containing 50 μM luminol and 10 μg/ml horseradish peroxidase (Sigma-Aldrich, USA) supplemented with or without 100 nM flg22. Luminescence was measured by a luminometer (GloMax-Multi Detection System, Promega, USA) for a period of 50 min with a signal integration time of 1 or 2 s.

### Diacylglycerol kinase activity assay

The in vitro DGK5 activity assay was performed as described previously with some modifications [13]. In brief, 2 µg of purified HIS-DGK5β or HIS-DGK5α proteins or 100 µg of cell lysates from 2-week-old WT or *cpl3-3* seedlings treated with/without 100 nM flg22 for 10 min were incubated with 1 μCi [^14^C]−1,2-dioleoyl-sn-glycerol (DOG, an unsaturated DAG analog, American Radiolabeled Chemicals Inc., USA) in a 250 µl reaction buffer, containing 40 mM Tris–HCl, pH 7.5, 5 mM MgCl_2_, 0.1 mM EGTA, 0.5 mM DTT, 1 mM sodium deoxycholate, 1 mM 3-[(3-cholamidopropyl) dimethylammonio]−1-propanesulfonate (CHAPS), 0.02% Triton X-100, and 10 μM ATP, for 30 min at 30 °C. The lipids DOG, dissolved in chloroform, were placed in 7 ml SCHOTT glass disposable reaction tubes with screw caps (Schott, Germany), dried under a stream of nitrogen vapor, resuspended in a solution of 1.47 mM sodium deoxycholate, and followed by sonication for 5 min (5 cycles of 10 s sonication and 10 s stop) using the Branson SFX 250 Sonifier (Emerson, USA) at 4 °C. The reaction was stopped by adding 750 µl chloroform/methanol (1:2) containing 1% HCl. Phospholipids were extracted by adding 1 ml of chloroform/methanol (1:1) to the solution, followed by the addition of 500 µl of a solution containing 1 M KCl and 0.2 M H_3_PO_4_. The mixture was thoroughly mixed by vortexing and then centrifuged at 2000 rpm for 5 min. The lower organic phase (lipids) was transferred to a new glass tube, dried under a stream of nitrogen vapor, and resuspended in 50 µl chloroform/methanol (2:1). The lipids were separated by TLC silica plates (Merck, USA) that had been activated by heating for 15 min at 110 °C. The plates were run in an acidic solvent system (chloroform/acetone/methanol/acetic acid/water, 40:15:14:12:8, v/v/v), and then put on paper towels to dry for 5–10 min. The radioactive lipid products were visualized by autoradiography using GE Typhoon FLA 9500 (GE Healthcare, USA).

### Quantification and statistical analysis

No statistical methods were used to predetermine sample size such as the number of genes. Blinding and randomization were not used. Data for quantification analyses are presented as mean ± s.e.m. for ROS detection or standard deviation (s.d.) for others. Statistical analyses were performed by two-sided Student’s *t*-test, one-way ANOVA followed by the Tukey’s test, Chow test [76], or Benjamini-Hochberg (BH) *t*-test for adjusted *p* value. The number of biologically independent replicates is indicated in figure legends. Exact *p* values are provided in the graphs.

## Supplementary Information

Additional file 1: Table S1a Summary of read counts in this study. Table S1b Summary of read counts in this study. Table S2a List of upregulated differentially expressed genes (DEGs) between mock and flg22 treated WT seedlings. Table S2b List of downregulated differentially expressed genes (DEGs) between mock and flg22 treated WT seedlings. Table S2c List of upregulated differentially expressed genes (DEGs) between mock and flg22 treated cpl3-3 seedlings. Table S2d List of downregulated differentially expressed genes (DEGs) between mock and flg22 treated cpl3-3 seedlings. Table S3a List of differentially alternatively spliced (DAS) genes between mock and flg22-treated WT seedlings. Table S3b List of differentially alternatively spliced (DAS) genes between mock and flg22-treated cpl3-3 seedlings. Table S4a List of upregulated differential transcript usages (DTUs) between mock and flg22-treated WT seedlings. Table S4b List of downregulated differential transcript usages (DTUs) between mock and flg22-treated WT seedlings. Table S4c List of upregulated differential transcript usages (DTUs) between mock and flg22-treated cpl3-3 seedlings. Table S4d List of downregulated differential transcript usages (DTUs) between mock and flg22-treated cpl3-3 seedlings. Table S5 GO terms and IDs used in this study. Table S6 List of basal differentially expressed genes (DEGs) between WT and cpl3-3 seedlings. Table S7 List of basal differentially alternatively spliced (DAS) genes between WT and cpl3-3 seedlings. Table S8a List of CPL3-dependent upregulated differentially expressed genes (DEGs) between mock and flg22-treated seedlings. Table S8b List of CPL3-dependent downregulated differentially expressed genes (DEGs) between mock and flg22-treated seedlings. Table S9a List of CPL3-dependent upregulated differential transcript usages (DTUs) between mock and flg22-treated seedlings. Table S9b List of CPL3 dependent downregulated differential transcript usages (DTUs) between mock and flg22-treated seedlings. Table S10 List of CPL3-dependent differentially alternatively spliced (DAS) genes between mock and flg22-treated seedlings. Table S11 Primers used in this study.

Additional file 2: Fig. S1 RNA-seq sample verification and global in-depth transcriptome analysis upon flg22 treatment in WT. Fig. S2 Characterization of the cpl3-3 mutant. Fig. S3 CPL3 preferentially regulates flg22-triggered alternative splicing rather than gene expression. Fig. S4 Additional examples of CPL3-dependent flg22-DASs. Fig. S5 Annotation and analysis of DGK5 splicing variants of DGK5β and DGK5α.

Additional file 3: Uncropped images.

## Data Availability

Sequence data in this study can be found in the Arabidopsis Genome Initiative under the following accession numbers: ACA12 (AT3G63380), BAK1 (AT4G33430), BIK1 (AT2G39660), BIR1 (AT5G48380), BTL2 (AT1G34420), CPL3 (AT2G33540), CRK29 (AT4G21410), DGK5 (AT2G20900), FRK1 (AT2G19190), MPK12 (AT2G46070), SERK4 (AT2G13790), EDS5 (AT4G39030), UBQ10 (AT4G05320), WRKY30 (AT5G24110), WTKY33 (AT2G38470). RNA-seq data in this study have been deposited in the Short Read Archive (SRA) database (https://www.ncbi.nlm.nih.gov/sra) with BioProject ID: PRJNA1124505 [77]. The source code and scripts in R for the analysis are available with XXX license at GitHub (https://github.com/Sung-Il-Kim/RNA-seq_WT-and-cpl3.git) in GitHub [78], and at Zenodo (https://doi.org/10.5281/zenodo.14890440) [79].
