# Supplementary material for: Global profiling of CPL3-mediated alternative splicing reveals regulatory mechanisms of DGK5 in plant immunity and phosphatidic acid homeostasis
Source: Genome Biol. 2025 Mar 21;26:65. doi: 10.1186/s13059-025-03529-2 (PMC11927175; doi:10.1186/s13059-025-03529-2)
Supplement: Supplementary file 2 — Additional file 2: Fig. S1 RNA-seq sample verification and global in-depth transcriptome analysis upon flg22 treatment in WT. Fig. S2 Characterization of the cpl3-3 mutant. Fig. S3 CPL3 preferentially regulates flg22-triggered alternative splicing rather than gene expression. Fig. S4 Additional examples of CPL3-dependent flg22-DASs. Fig. S5 Annotation and analysis of DGK5 splicing variants of DGK5β and DGK5α. [file 13059_2025_3529_MOESM2_ESM.pptx]

## Slide 1
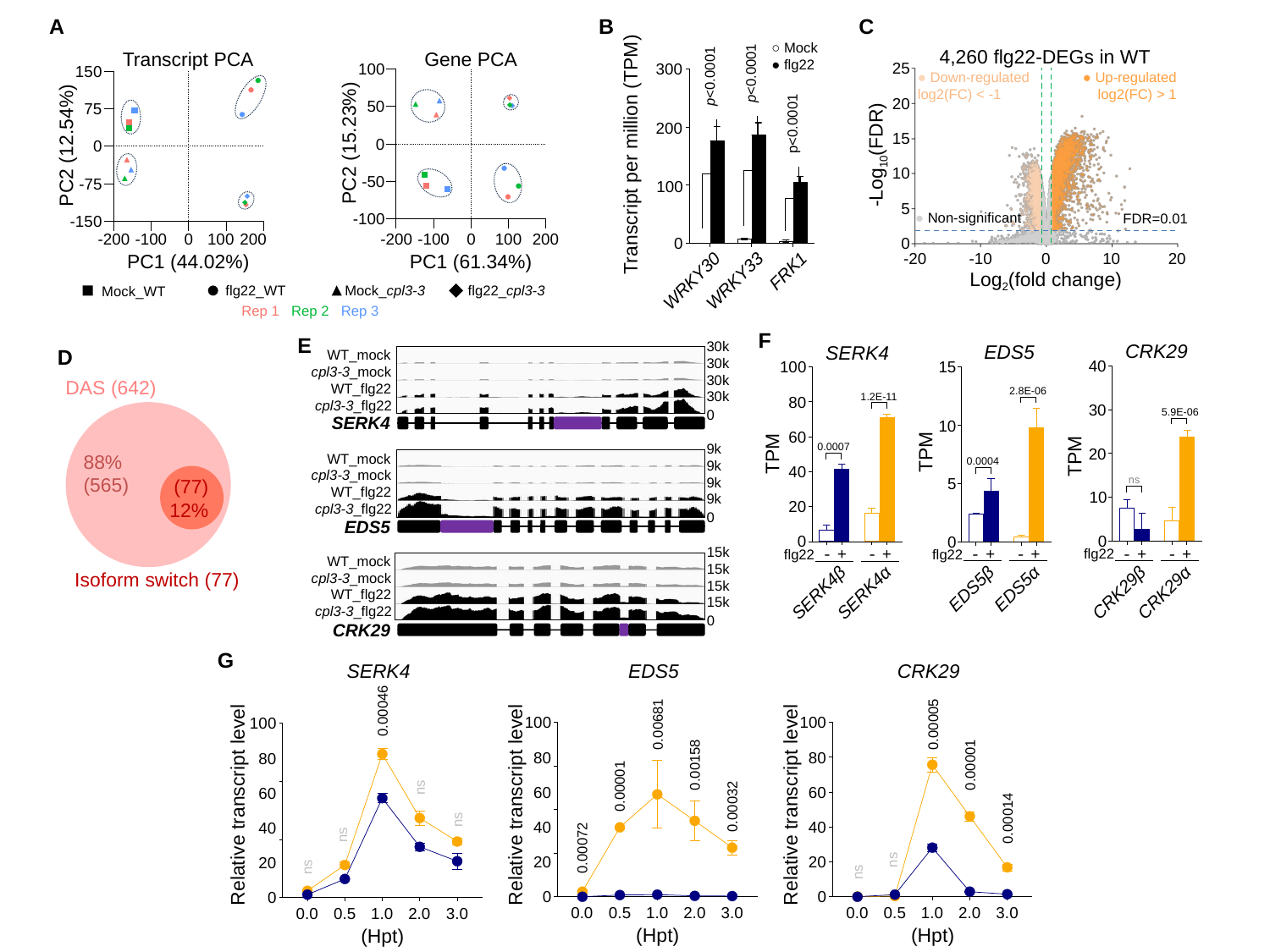

A
B
C
○ Mock
● flg22
p<0.0001
300
p<0.0001
p<0.0001
200
Transcript per million (TPM)
100
0
FRK1
WRKY33
WRKY30
4,260 flg22-DEGs in WT
25
● Up-regulated
log2(FC) > 1
● Down-regulated
log2(FC) < -1
20
15
-Log10(FDR)
10
5
● Non-significant
FDR=0.01
0
-20
-10
0
10
20
Log2(fold change)
Transcript PCA
Gene PCA
100
150
50
75
PC2 (15.23%)
PC2 (12.54%)
0
0
-50
-75
-100
-150
-200
-100
0
200
-200
-100
0
100
200
100
PC1 (44.02%)
PC1 (61.34%)
flg22_WT
Mock_cpl3-3
flg22_cpl3-3
Mock_WT
Rep 1 Rep 2 Rep 3
F
CRK29
40
5.9E-06
30
20
TPM
ns
10
0
-
+
-
+
flg22
CRK29α
CRK29β
EDS5
15
2.8E-06
10
TPM
0.0004
5
0
-
+
-
+
flg22
EDS5α
EDS5β
SERK4
100
1.2E-11
80
60
0.0007
TPM
40
20
0
-
+
-
+
flg22
SERK4α
SERK4β
E
D
DAS (642)
88%
(565)
(77)
12%
Isoform switch (77)
30k
30k
30k
30k
0
WT_mock
cpl3-3_mock
WT_flg22
cpl3-3_flg22
SERK4
9k
9k
9k
9k
0
WT_mock
cpl3-3_mock
WT_flg22
cpl3-3_flg22
EDS5
15k
15k
15k
15k
0
WT_mock
cpl3-3_mock
WT_flg22
cpl3-3_flg22
CRK29
G
SERK4
0.00046
100
80
ns
60
Relative transcript level
ns
ns
40
ns
20
0
0.0
0.5
1.0
2.0
3.0
(Hpt)
EDS5
0.00681
100
80
0.00158
0.00001
60
0.00032
Relative transcript level
40
0.00072
20
0
0.0
0.5
1.0
2.0
3.0
(Hpt)
CRK29
0.00005
100
0.00001
80
60
Relative transcript level
0.00014
40
ns
ns
20
0
0.0
0.5
1.0
2.0
3.0
(Hpt)

## Slide 2
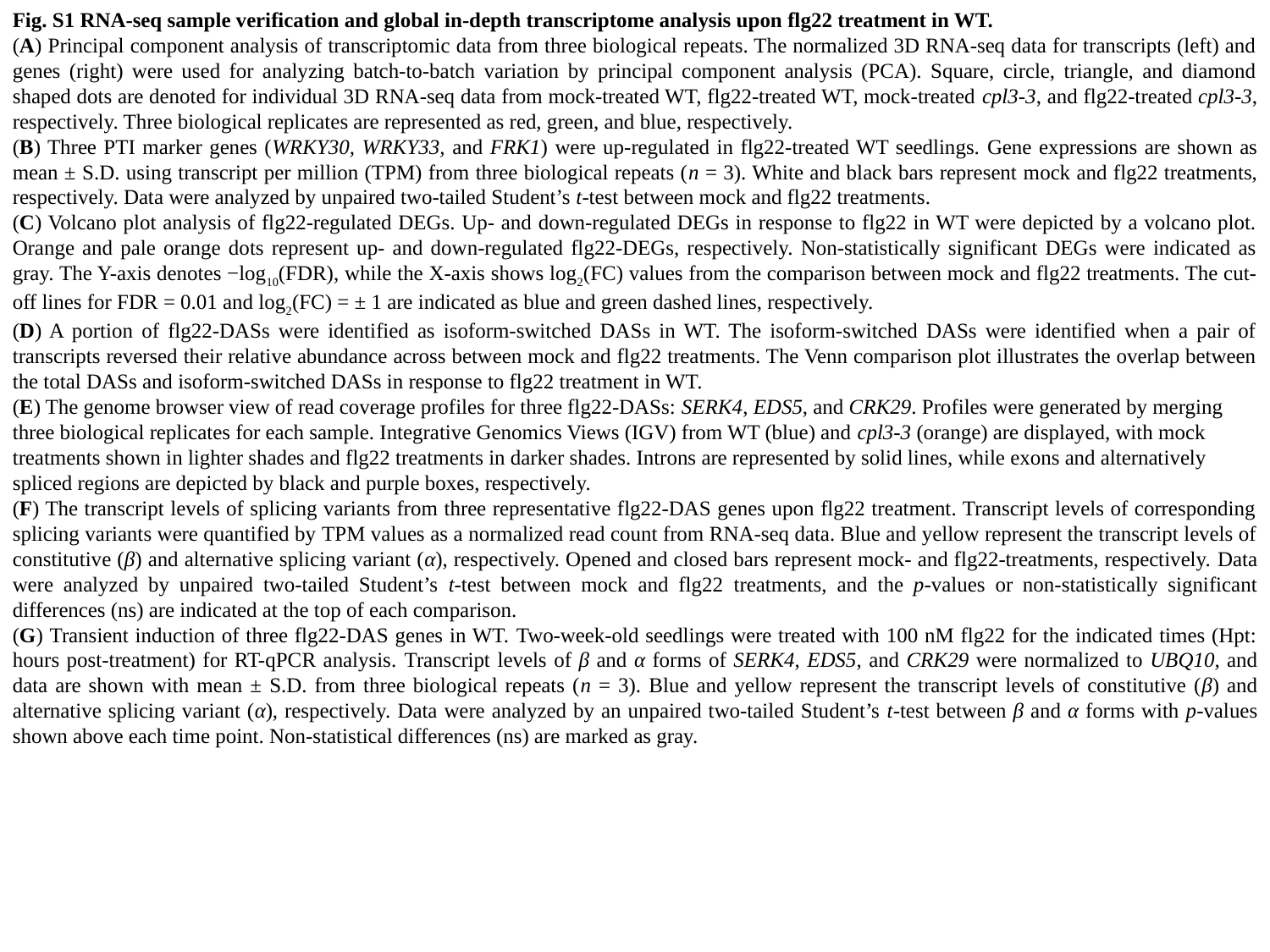

Fig. S1 RNA-seq sample verification and global in-depth transcriptome analysis upon flg22 treatment in WT.
(A) Principal component analysis of transcriptomic data from three biological repeats. The normalized 3D RNA-seq data for transcripts (left) and genes (right) were used for analyzing batch-to-batch variation by principal component analysis (PCA). Square, circle, triangle, and diamond shaped dots are denoted for individual 3D RNA-seq data from mock-treated WT, flg22-treated WT, mock-treated cpl3-3, and flg22-treated cpl3-3, respectively. Three biological replicates are represented as red, green, and blue, respectively.
(B) Three PTI marker genes (WRKY30, WRKY33, and FRK1) were up-regulated in flg22-treated WT seedlings. Gene expressions are shown as mean ± S.D. using transcript per million (TPM) from three biological repeats (n = 3). White and black bars represent mock and flg22 treatments, respectively. Data were analyzed by unpaired two-tailed Student’s t-test between mock and flg22 treatments.
(C) Volcano plot analysis of flg22-regulated DEGs. Up- and down-regulated DEGs in response to flg22 in WT were depicted by a volcano plot. Orange and pale orange dots represent up- and down-regulated flg22-DEGs, respectively. Non-statistically significant DEGs were indicated as gray. The Y-axis denotes −log10(FDR), while the X-axis shows log2(FC) values from the comparison between mock and flg22 treatments. The cut-off lines for FDR = 0.01 and log2(FC) = ± 1 are indicated as blue and green dashed lines, respectively.
(D) A portion of flg22-DASs were identified as isoform-switched DASs in WT. The isoform-switched DASs were identified when a pair of transcripts reversed their relative abundance across between mock and flg22 treatments. The Venn comparison plot illustrates the overlap between the total DASs and isoform-switched DASs in response to flg22 treatment in WT.
(E) The genome browser view of read coverage profiles for three flg22-DASs: SERK4, EDS5, and CRK29. Profiles were generated by merging three biological replicates for each sample. Integrative Genomics Views (IGV) from WT (blue) and cpl3-3 (orange) are displayed, with mock treatments shown in lighter shades and flg22 treatments in darker shades. Introns are represented by solid lines, while exons and alternatively spliced regions are depicted by black and purple boxes, respectively.
(F) The transcript levels of splicing variants from three representative flg22-DAS genes upon flg22 treatment. Transcript levels of corresponding splicing variants were quantified by TPM values as a normalized read count from RNA-seq data. Blue and yellow represent the transcript levels of constitutive (β) and alternative splicing variant (α), respectively. Opened and closed bars represent mock- and flg22-treatments, respectively. Data were analyzed by unpaired two-tailed Student’s t-test between mock and flg22 treatments, and the p-values or non-statistically significant differences (ns) are indicated at the top of each comparison.
(G) Transient induction of three flg22-DAS genes in WT. Two-week-old seedlings were treated with 100 nM flg22 for the indicated times (Hpt: hours post-treatment) for RT-qPCR analysis. Transcript levels of β and α forms of SERK4, EDS5, and CRK29 were normalized to UBQ10, and data are shown with mean ± S.D. from three biological repeats (n = 3). Blue and yellow represent the transcript levels of constitutive (β) and alternative splicing variant (α), respectively. Data were analyzed by an unpaired two-tailed Student’s t-test between β and α forms with p-values shown above each time point. Non-statistical differences (ns) are marked as gray.

## Slide 3
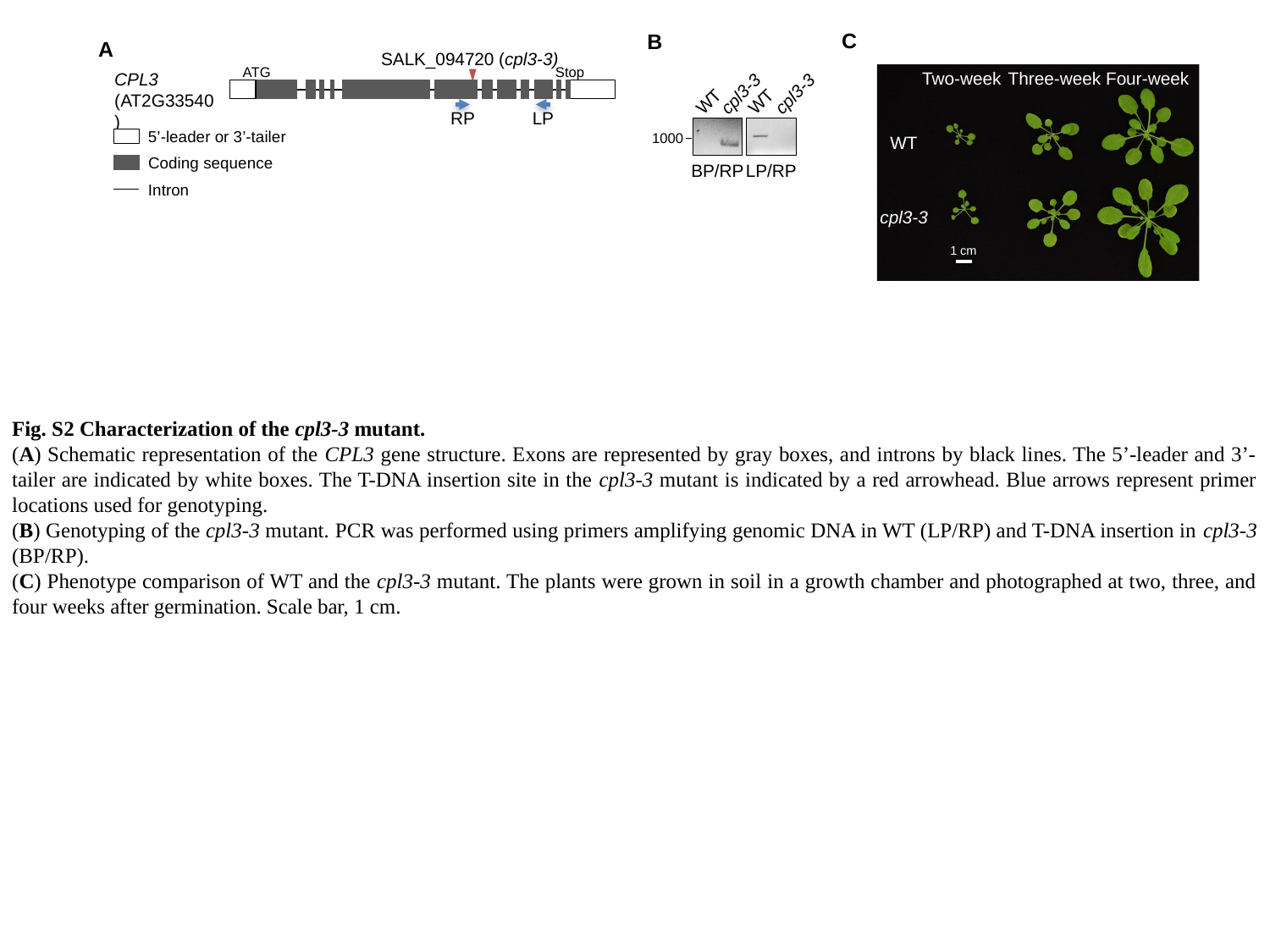

C
B
A
SALK_094720 (cpl3-3)
ATG
Stop
RP
LP
Two-week
Three-week
Four-week
WT
cpl3-3
1 cm
CPL3 (AT2G33540)
cpl3-3
cpl3-3
WT
WT
1000
BP/RP
LP/RP
5’-leader or 3’-tailer
Coding sequence
Intron
Fig. S2 Characterization of the cpl3-3 mutant.
(A) Schematic representation of the CPL3 gene structure. Exons are represented by gray boxes, and introns by black lines. The 5’-leader and 3’-tailer are indicated by white boxes. The T-DNA insertion site in the cpl3-3 mutant is indicated by a red arrowhead. Blue arrows represent primer locations used for genotyping.
(B) Genotyping of the cpl3-3 mutant. PCR was performed using primers amplifying genomic DNA in WT (LP/RP) and T-DNA insertion in cpl3-3 (BP/RP).
(C) Phenotype comparison of WT and the cpl3-3 mutant. The plants were grown in soil in a growth chamber and photographed at two, three, and four weeks after germination. Scale bar, 1 cm.

## Slide 4
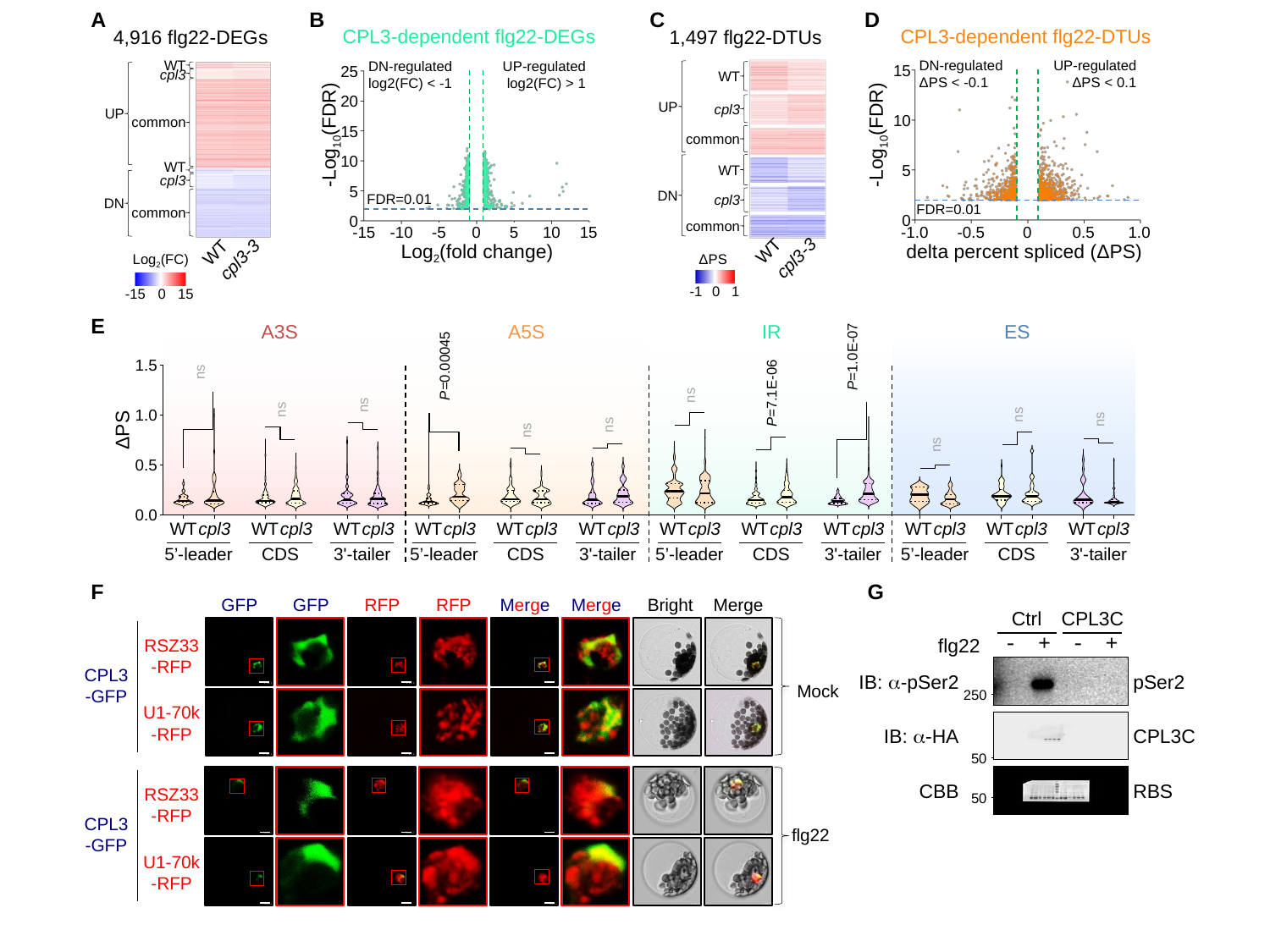

B
D
A
C
1,497 flg22-DTUs
WT
UP
cpl3
common
WT
DN
cpl3
common
WT
cpl3-3
ΔPS
-1
0
1
4,916 flg22-DEGs
CPL3-dependent flg22-DTUs
CPL3-dependent flg22-DEGs
UP-regulated
log2(FC) > 1
DN-regulated
log2(FC) < -1
25
20
15
10
5
FDR=0.01
0
WT
cpl3
UP-regulated
ΔPS < 0.1
DN-regulated
ΔPS < -0.1
15
UP
common
10
-Log10(FDR)
-Log10(FDR)
WT
5
cpl3
DN
common
FDR=0.01
0
-15
-10
-5
0
5
10
15
-1.0
-0.5
0
0.5
1.0
Log2(fold change)
delta percent spliced (ΔPS)
WT
cpl3-3
Log2(FC)
-15
0
15
E
A3S
A5S
IR
ES
ns
P=1.0E-07
P=0.00045
ns
1.5
ns
ns
ns
P=7.1E-06
ns
ns
ns
ns
1.0
ΔPS
0.5
0.0
WT
cpl3
WT
cpl3
WT
cpl3
WT
cpl3
WT
cpl3
WT
cpl3
WT
cpl3
WT
cpl3
WT
cpl3
WT
cpl3
WT
cpl3
WT
cpl3
5’-leader
CDS
3'-tailer
5’-leader
CDS
3'-tailer
5’-leader
CDS
3'-tailer
5’-leader
CDS
3'-tailer
F
G
GFP
GFP
RFP
RFP
Merge
Merge
Bright
Merge
Ctrl
CPL3C
flg22
RSZ33
-RFP
| - | + | - | + |
| --- | --- | --- | --- |
CPL3
-GFP
IB: α-pSer2
pSer2
Mock
250
U1-70k
-RFP
IB: α-HA
CPL3C
50
CBB
RBS
RSZ33
-RFP
50
CPL3
-GFP
flg22
U1-70k
-RFP

## Slide 5
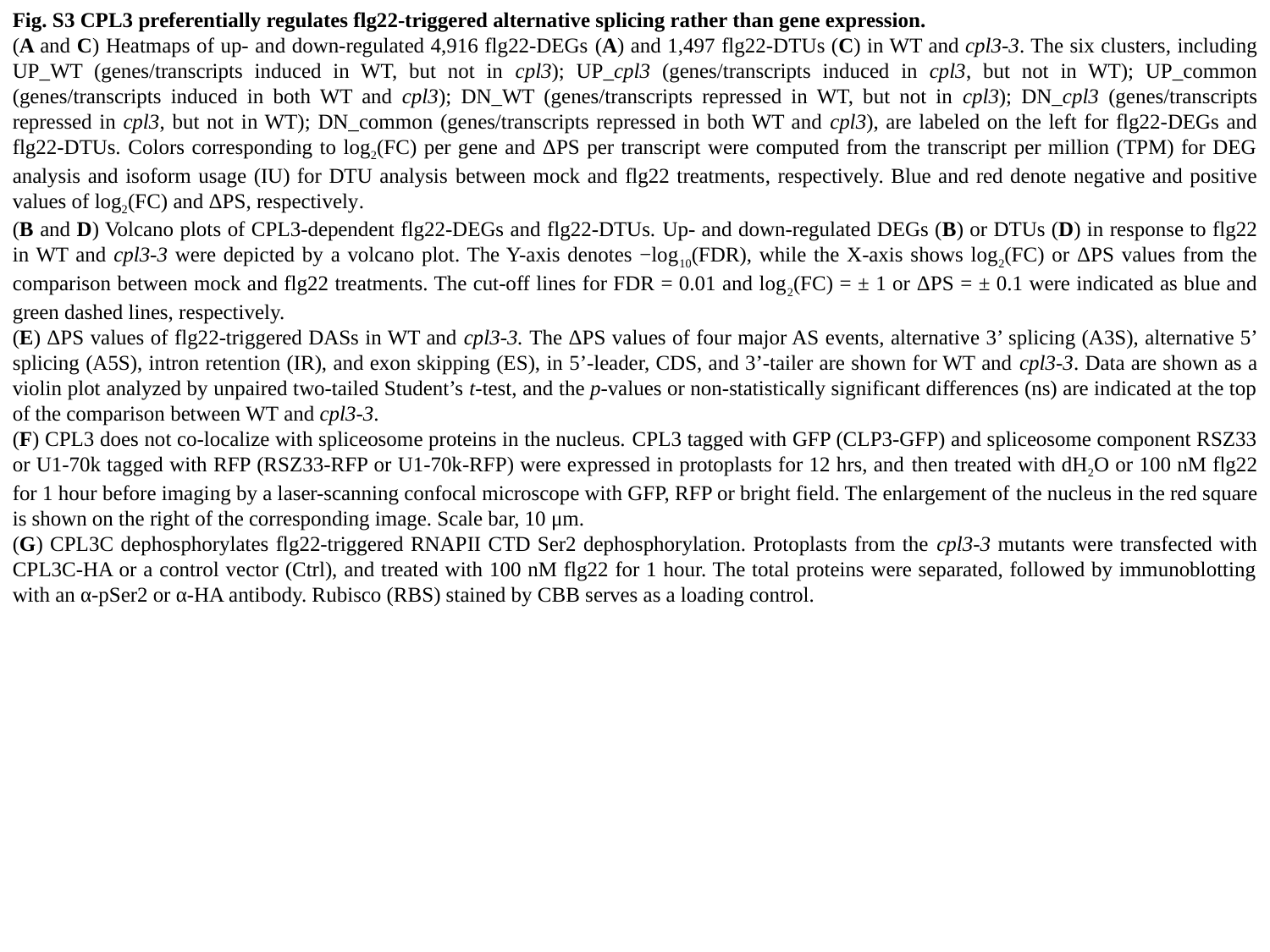

Fig. S3 CPL3 preferentially regulates flg22-triggered alternative splicing rather than gene expression.
(A and C) Heatmaps of up- and down-regulated 4,916 flg22-DEGs (A) and 1,497 flg22-DTUs (C) in WT and cpl3-3. The six clusters, including UP_WT (genes/transcripts induced in WT, but not in cpl3); UP_cpl3 (genes/transcripts induced in cpl3, but not in WT); UP_common (genes/transcripts induced in both WT and cpl3); DN_WT (genes/transcripts repressed in WT, but not in cpl3); DN_cpl3 (genes/transcripts repressed in cpl3, but not in WT); DN_common (genes/transcripts repressed in both WT and cpl3), are labeled on the left for flg22-DEGs and flg22-DTUs. Colors corresponding to log2(FC) per gene and ΔPS per transcript were computed from the transcript per million (TPM) for DEG analysis and isoform usage (IU) for DTU analysis between mock and flg22 treatments, respectively. Blue and red denote negative and positive values of log2(FC) and ΔPS, respectively.
(B and D) Volcano plots of CPL3-dependent flg22-DEGs and flg22-DTUs. Up- and down-regulated DEGs (B) or DTUs (D) in response to flg22 in WT and cpl3-3 were depicted by a volcano plot. The Y-axis denotes −log10(FDR), while the X-axis shows log2(FC) or ΔPS values from the comparison between mock and flg22 treatments. The cut-off lines for FDR = 0.01 and log2(FC) = ± 1 or ΔPS = ± 0.1 were indicated as blue and green dashed lines, respectively.
(E) ΔPS values of flg22-triggered DASs in WT and cpl3-3. The ΔPS values of four major AS events, alternative 3’ splicing (A3S), alternative 5’ splicing (A5S), intron retention (IR), and exon skipping (ES), in 5’-leader, CDS, and 3’-tailer are shown for WT and cpl3-3. Data are shown as a violin plot analyzed by unpaired two-tailed Student’s t-test, and the p-values or non-statistically significant differences (ns) are indicated at the top of the comparison between WT and cpl3-3.
(F) CPL3 does not co-localize with spliceosome proteins in the nucleus. CPL3 tagged with GFP (CLP3-GFP) and spliceosome component RSZ33 or U1-70k tagged with RFP (RSZ33-RFP or U1-70k-RFP) were expressed in protoplasts for 12 hrs, and then treated with dH2O or 100 nM flg22 for 1 hour before imaging by a laser-scanning confocal microscope with GFP, RFP or bright field. The enlargement of the nucleus in the red square is shown on the right of the corresponding image. Scale bar, 10 μm.
(G) CPL3C dephosphorylates flg22-triggered RNAPII CTD Ser2 dephosphorylation. Protoplasts from the cpl3-3 mutants were transfected with CPL3C-HA or a control vector (Ctrl), and treated with 100 nM flg22 for 1 hour. The total proteins were separated, followed by immunoblotting with an α-pSer2 or α-HA antibody. Rubisco (RBS) stained by CBB serves as a loading control.

## Slide 6
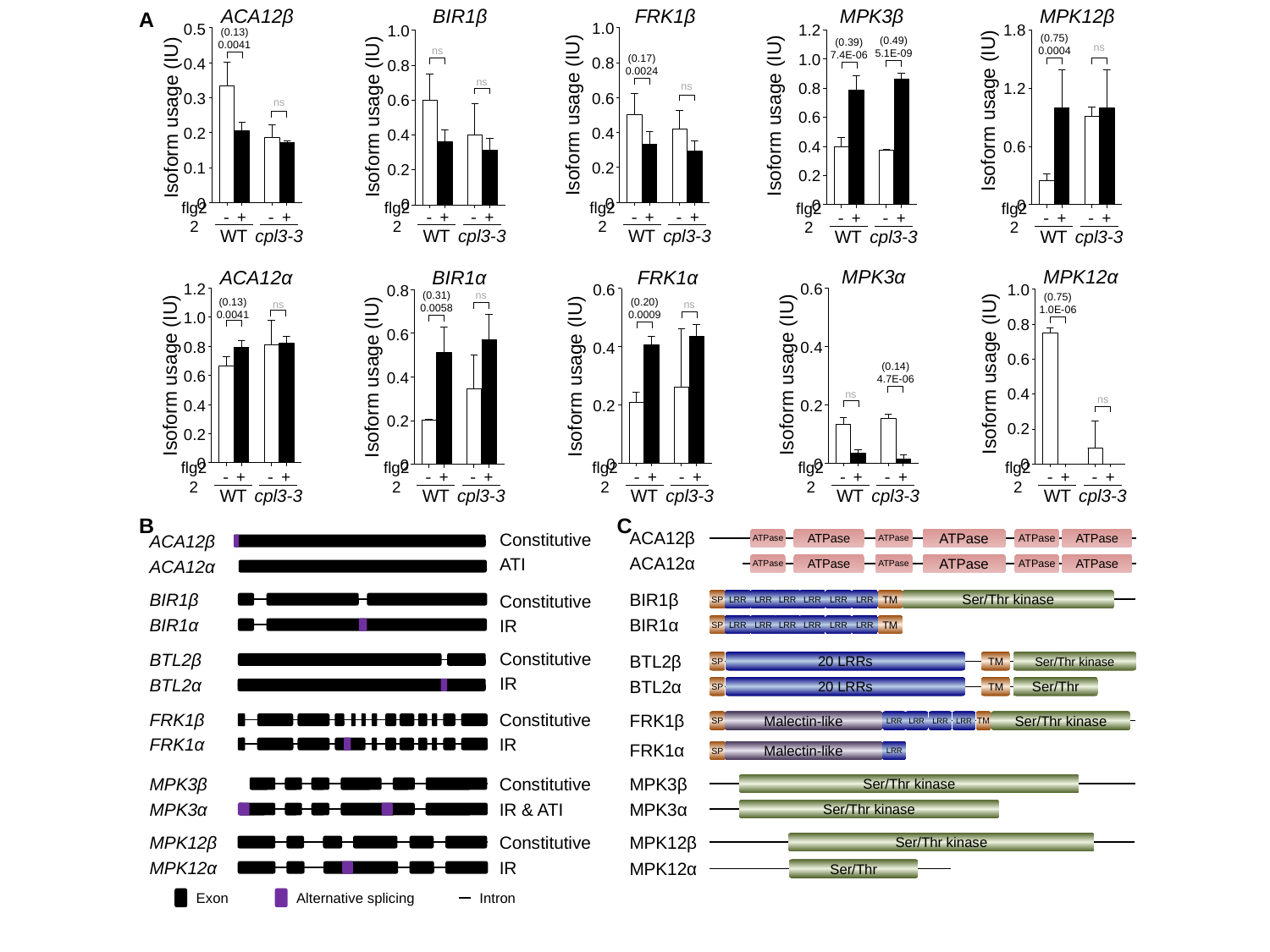

A
ACA12β
BIR1β
FRK1β
MPK3β
MPK12β
1.0
(0.13)
0.0041
0.5
1.2
1.8
1.0
(0.75)
0.0004
(0.49)
5.1E-09
(0.39)
7.4E-06
ns
ns
(0.17)
0.0024
1.0
0.8
0.4
0.8
ns
ns
0.8
1.2
0.6
0.3
ns
0.6
Isoform usage (IU)
Isoform usage (IU)
Isoform usage (IU)
Isoform usage (IU)
Isoform usage (IU)
0.6
0.4
0.2
0.4
0.4
0.6
0.2
0.1
0.2
0.2
0
0
0
0
0
flg22
-
+
-
+
flg22
-
+
-
+
flg22
-
+
-
+
flg22
-
+
-
+
flg22
-
+
-
+
WT
cpl3-3
WT
cpl3-3
WT
cpl3-3
WT
cpl3-3
WT
cpl3-3
MPK3α
MPK12α
ACA12α
BIR1α
FRK1α
1.2
0.6
0.6
1.0
0.8
(0.31)
0.0058
ns
(0.75)
1.0E-06
(0.13)
0.0041
(0.20)
0.0009
ns
ns
1.0
0.8
0.6
0.8
0.4
0.4
0.6
(0.14)
4.7E-06
Isoform usage (IU)
Isoform usage (IU)
Isoform usage (IU)
Isoform usage (IU)
Isoform usage (IU)
0.6
0.4
ns
0.4
ns
0.4
0.2
0.2
0.2
0.2
0.2
0
0
0
0
0
flg22
-
+
-
+
flg22
-
+
-
+
flg22
-
+
-
+
flg22
-
+
-
+
flg22
-
+
-
+
WT
cpl3-3
WT
cpl3-3
WT
cpl3-3
WT
cpl3-3
WT
cpl3-3
B
C
ACA12β
Constitutive
ACA12β
ATPase
ATPase
ATPase
ATPase
ATPase
ATPase
ATPase
ATPase
ATPase
ATPase
ATPase
ATPase
ACA12α
ATI
ACA12α
BIR1β
BIR1β
Constitutive
SP
LRR
LRR
LRR
LRR
LRR
LRR
TM
Ser/Thr kinase
SP
LRR
LRR
LRR
LRR
LRR
LRR
TM
BIR1α
BIR1α
IR
Constitutive
BTL2β
BTL2β
SP
20 LRRs
TM
Ser/Thr kinase
SP
20 LRRs
TM
Ser/Thr
IR
BTL2α
BTL2α
FRK1β
Constitutive
FRK1β
LRR
LRR
LRR
LRR
SP
Malectin-like
TM
Ser/Thr kinase
LRR
SP
Malectin-like
FRK1α
IR
FRK1α
MPK3β
Constitutive
MPK3β
Ser/Thr kinase
Ser/Thr kinase
MPK3α
IR & ATI
MPK3α
MPK12β
Constitutive
MPK12β
Ser/Thr kinase
Ser/Thr
MPK12α
IR
MPK12α
Intron
Exon
Alternative splicing

## Slide 7
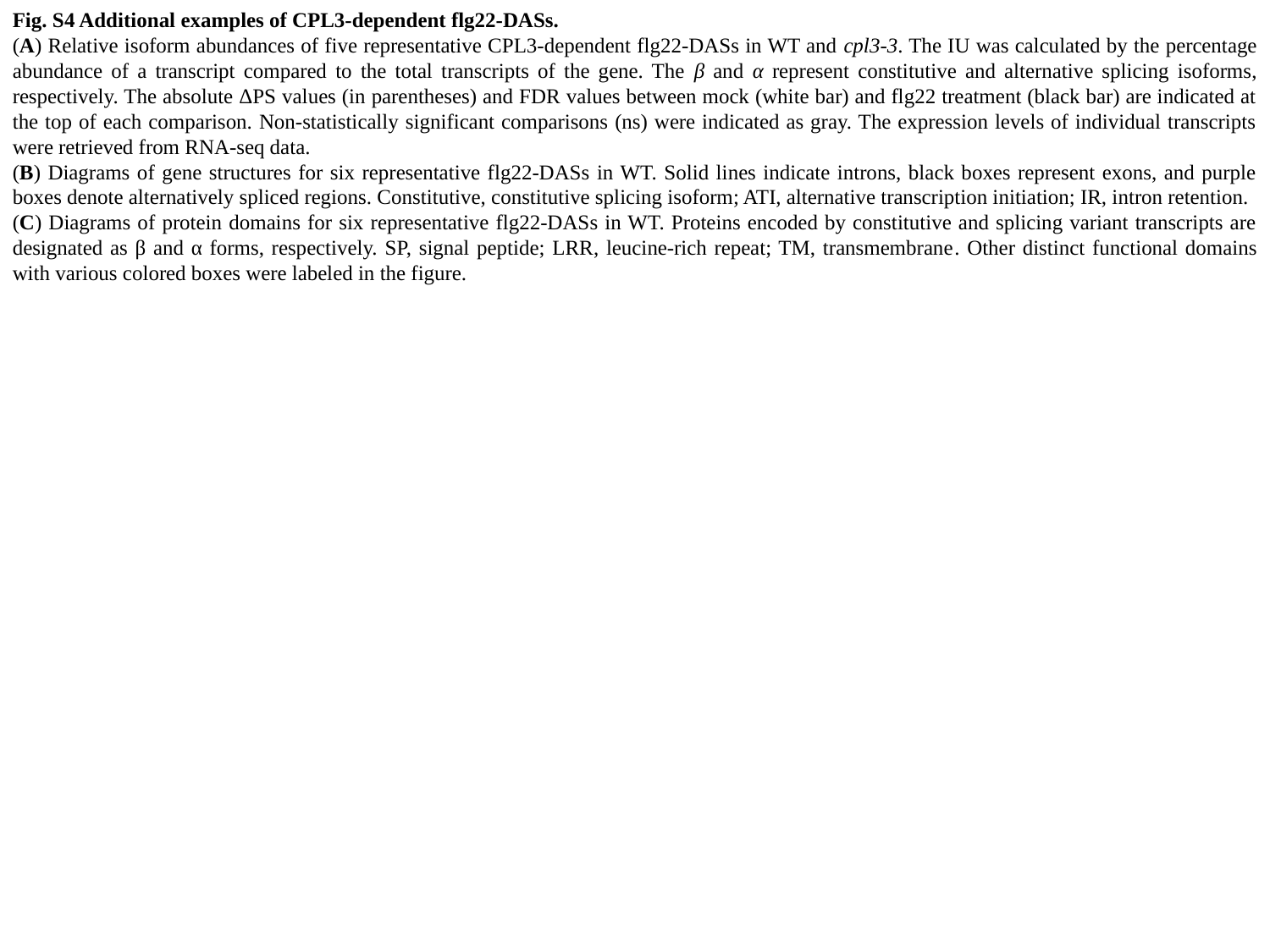

Fig. S4 Additional examples of CPL3-dependent flg22-DASs.
(A) Relative isoform abundances of five representative CPL3-dependent flg22-DASs in WT and cpl3-3. The IU was calculated by the percentage abundance of a transcript compared to the total transcripts of the gene. The β and α represent constitutive and alternative splicing isoforms, respectively. The absolute ΔPS values (in parentheses) and FDR values between mock (white bar) and flg22 treatment (black bar) are indicated at the top of each comparison. Non-statistically significant comparisons (ns) were indicated as gray. The expression levels of individual transcripts were retrieved from RNA-seq data.
(B) Diagrams of gene structures for six representative flg22-DASs in WT. Solid lines indicate introns, black boxes represent exons, and purple boxes denote alternatively spliced regions. Constitutive, constitutive splicing isoform; ATI, alternative transcription initiation; IR, intron retention.
(C) Diagrams of protein domains for six representative flg22-DASs in WT. Proteins encoded by constitutive and splicing variant transcripts are designated as β and α forms, respectively. SP, signal peptide; LRR, leucine-rich repeat; TM, transmembrane. Other distinct functional domains with various colored boxes were labeled in the figure.

## Slide 8
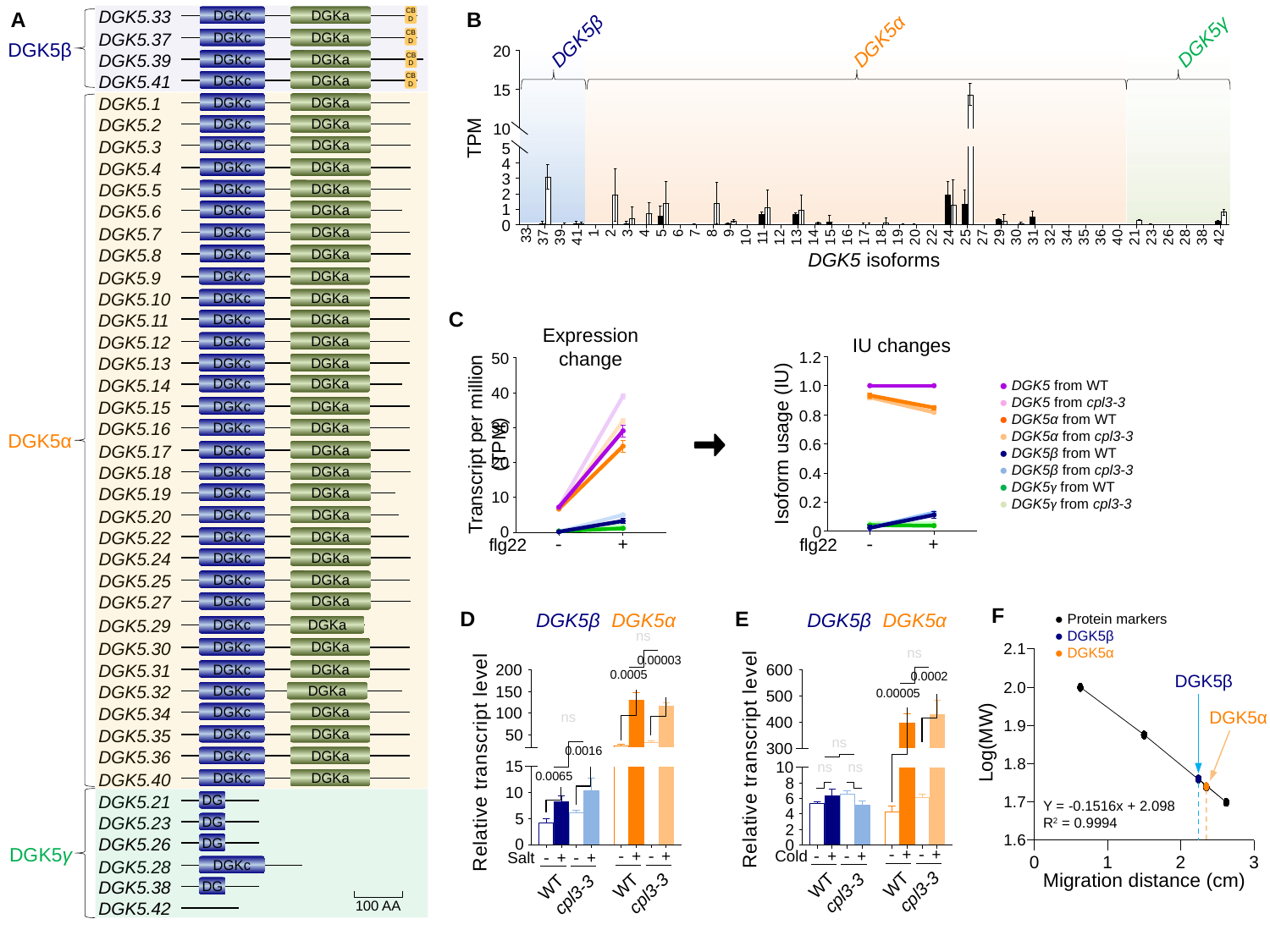

DGK5.33
A
B
CBD
DGKc
DGKa
DGK5.37
DGK5β
DGK5α
DGK5γ
CBD
DGKc
DGKa
DGK5β
20
DGK5.39
DGKc
DGKa
CBD
DGK5.41
CBD
DGKc
DGKa
15
DGK5.1
DGKc
DGKa
DGK5.2
DGKc
DGKa
10
TPM
DGK5.3
DGKc
DGKa
5
DGK5.4
4
DGKc
DGKa
3
DGK5.5
DGKc
DGKa
2
DGK5.6
1
DGKc
DGKa
0
DGK5.7
DGKc
DGKa
33
12
37
39
41
1
2
3
4
5
6
7
8
9
10
11
13
14
15
16
17
18
19
20
22
24
25
27
29
30
31
32
34
35
36
40
21
23
26
28
38
42
DGK5.8
DGKc
DGKa
DGK5 isoforms
DGK5.9
DGKc
DGKa
DGK5.10
DGKc
DGKa
C
DGK5.11
DGKc
DGKa
Expression change
DGK5.12
DGKc
DGKa
IU changes
DGK5.13
1.2
50
DGKc
DGKa
DGK5.14
● DGK5 from WT
● DGK5 from cpl3-3
● DGK5α from WT
● DGK5α from cpl3-3
● DGK5β from WT
● DGK5β from cpl3-3
● DGK5γ from WT
● DGK5γ from cpl3-3
DGKc
DGKa
1.0
40
DGK5.15
DGKc
DGKa
0.8
DGK5.16
30
DGKc
DGKa
DGK5α
Isoform usage (IU)
Transcript per million (TPM)
0.6
DGK5.17
DGKc
DGKa
20
DGK5.18
DGKc
DGKa
0.4
DGK5.19
DGKc
DGKa
10
0.2
DGK5.20
DGKc
DGKa
DGK5.22
0
0
flg22
flg22
DGKc
DGKa
-
+
-
+
DGK5.24
DGKc
DGKa
DGK5.25
DGKc
DGKa
2.1
2.0
1.9
Log(MW)
1.8
1.7
1.6
0
1
2
3
Migration distance (cm)
DGK5β
DGK5α
Y = -0.1516x + 2.098
R2 = 0.9994
F
● Protein markers
● DGK5β
● DGK5α
DGK5.27
DGKc
DGKa
D
E
DGK5β
DGK5α
DGK5β
DGK5α
DGK5.29
DGKc
DGKa
ns
DGK5.30
ns
DGKc
DGKa
0.00003
l
e
v
e
l
t
p
i
r
c
s
n
a
r
t
e
v
i
t
a
l
e
R
DGK5.31
l
e
v
e
l
t
p
i
r
c
s
n
a
r
t
e
v
i
t
a
l
e
R
200
DGKc
DGKa
600
0.0005
0.0002
DGK5.32
0.00005
150
DGKc
DGKa
500
DGK5.34
ns
DGKc
DGKa
100
400
DGK5.35
50
DGKc
DGKa
ns
0.0016
300
DGK5.36
DGKc
DGKa
ns
ns
15
10
0.0065
DGK5.40
DGKc
DGKa
8
10
DGK5.21
6
DG
4
DGK5.23
5
DG
2
DGK5.26
DG
0
0
DGK5γ
-
+
-
+
-
+
-
+
-
+
-
+
-
+
-
+
Cold
Salt
DGK5.28
DGKc
DGK5.38
DG
WT
cpl3-3
WT
cpl3-3
WT
cpl3-3
WT
cpl3-3
100 AA
DGK5.42

## Slide 9
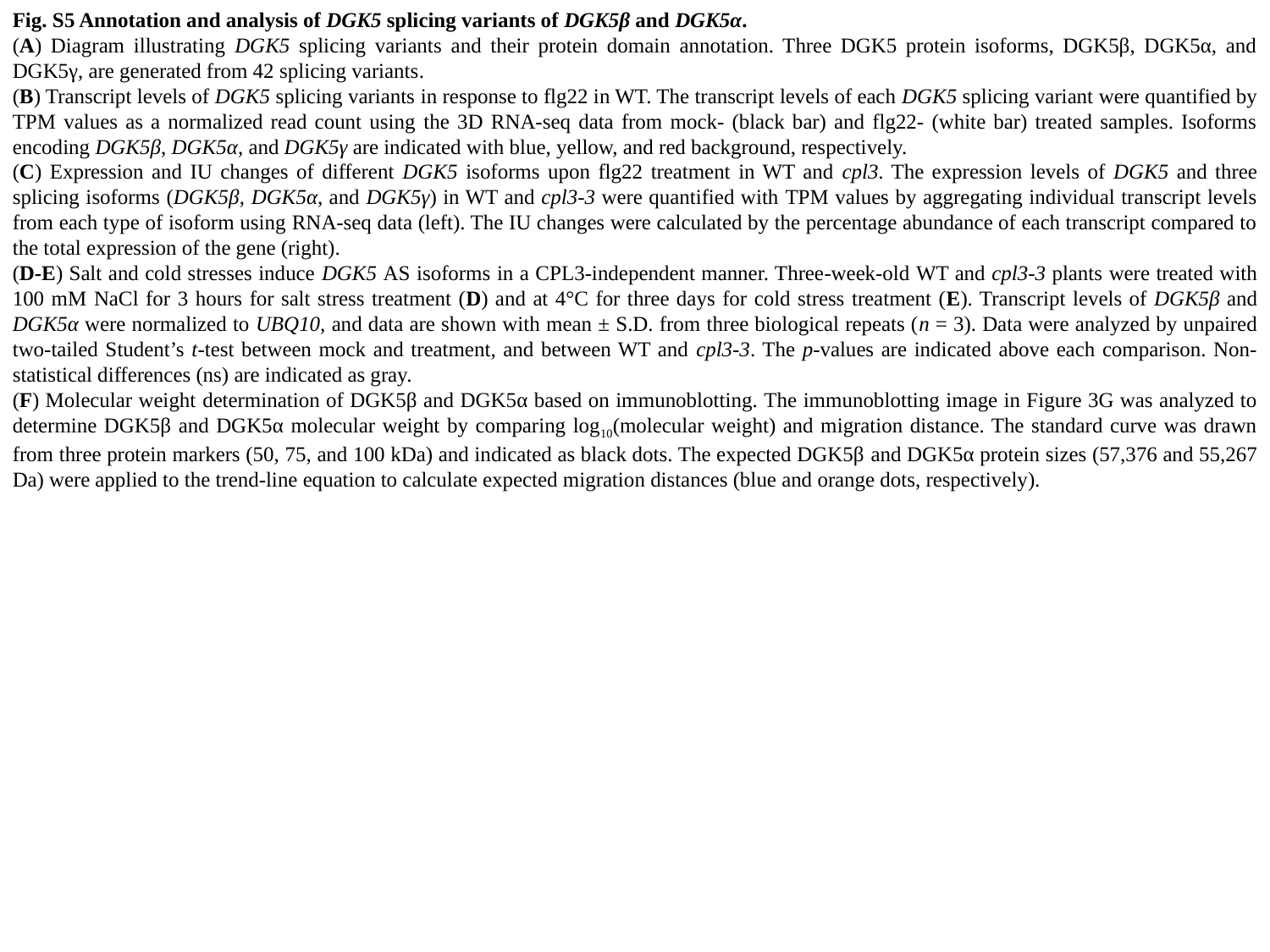

Fig. S5 Annotation and analysis of DGK5 splicing variants of DGK5β and DGK5α.
(A) Diagram illustrating DGK5 splicing variants and their protein domain annotation. Three DGK5 protein isoforms, DGK5β, DGK5α, and DGK5γ, are generated from 42 splicing variants.
(B) Transcript levels of DGK5 splicing variants in response to flg22 in WT. The transcript levels of each DGK5 splicing variant were quantified by TPM values as a normalized read count using the 3D RNA-seq data from mock- (black bar) and flg22- (white bar) treated samples. Isoforms encoding DGK5β, DGK5α, and DGK5γ are indicated with blue, yellow, and red background, respectively.
(C) Expression and IU changes of different DGK5 isoforms upon flg22 treatment in WT and cpl3. The expression levels of DGK5 and three splicing isoforms (DGK5β, DGK5α, and DGK5γ) in WT and cpl3-3 were quantified with TPM values by aggregating individual transcript levels from each type of isoform using RNA-seq data (left). The IU changes were calculated by the percentage abundance of each transcript compared to the total expression of the gene (right).
(D-E) Salt and cold stresses induce DGK5 AS isoforms in a CPL3-independent manner. Three-week-old WT and cpl3-3 plants were treated with 100 mM NaCl for 3 hours for salt stress treatment (D) and at 4°C for three days for cold stress treatment (E). Transcript levels of DGK5β and DGK5α were normalized to UBQ10, and data are shown with mean ± S.D. from three biological repeats (n = 3). Data were analyzed by unpaired two-tailed Student’s t-test between mock and treatment, and between WT and cpl3-3. The p-values are indicated above each comparison. Non-statistical differences (ns) are indicated as gray.
(F) Molecular weight determination of DGK5β and DGK5α based on immunoblotting. The immunoblotting image in Figure 3G was analyzed to determine DGK5β and DGK5α molecular weight by comparing log10(molecular weight) and migration distance. The standard curve was drawn from three protein markers (50, 75, and 100 kDa) and indicated as black dots. The expected DGK5β and DGK5α protein sizes (57,376 and 55,267 Da) were applied to the trend-line equation to calculate expected migration distances (blue and orange dots, respectively).
