# Supplementary material for: Global profiling of CPL3-mediated alternative splicing reveals regulatory mechanisms of DGK5 in plant immunity and phosphatidic acid homeostasis
Source: Genome Biol. 2025 Mar 21;26:65. doi: 10.1186/s13059-025-03529-2 (PMC11927175; doi:10.1186/s13059-025-03529-2)
Supplement: Supplementary file 3 — Additional file 3: Uncropped images. [file 13059_2025_3529_MOESM3_ESM.pptx]

## Slide 1
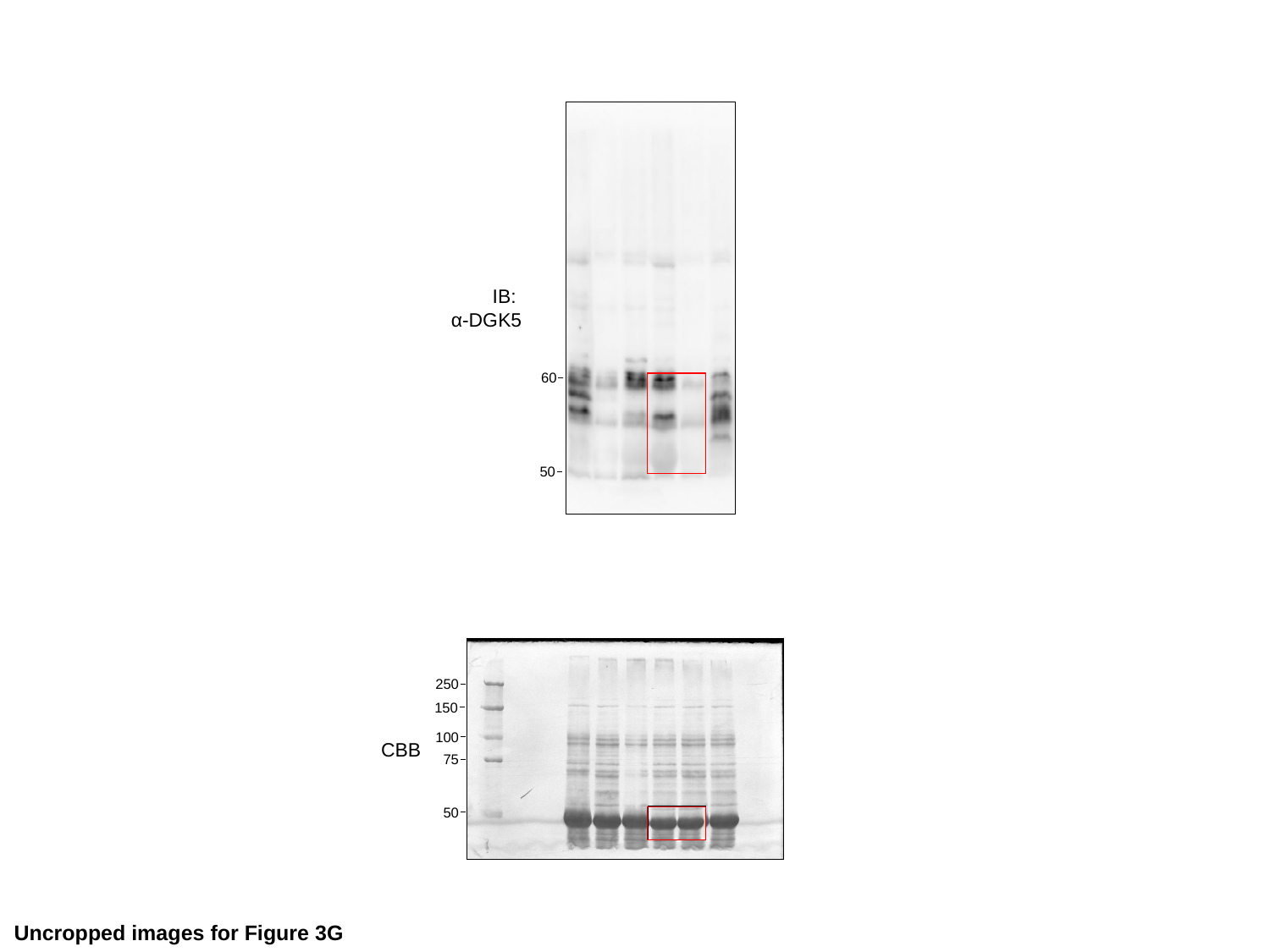

IB:
α-DGK5
60
50
250
150
100
CBB
75
50
Uncropped images for Figure 3G

## Slide 2
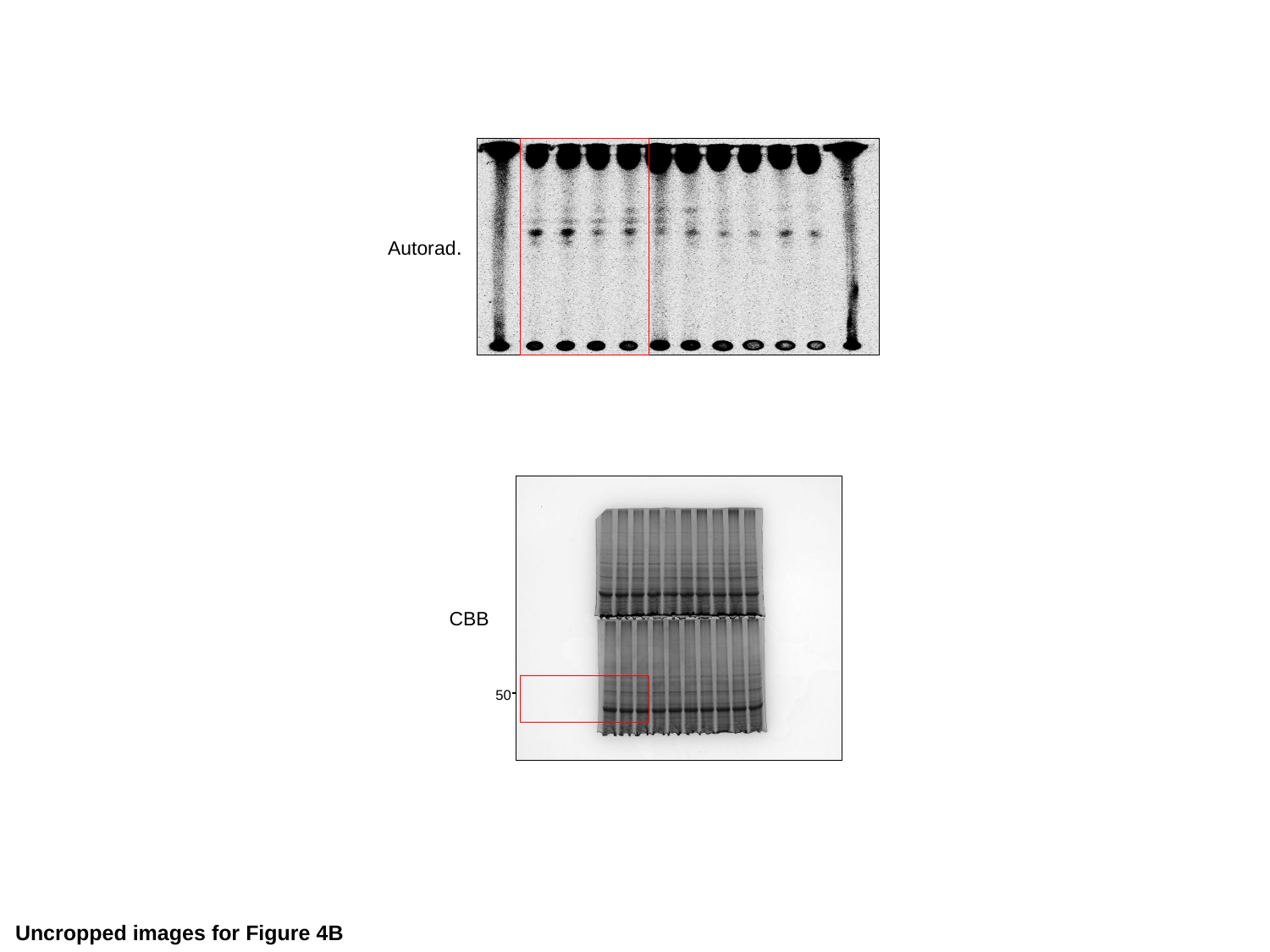

Autorad.
CBB
50
Uncropped images for Figure 4B

## Slide 3
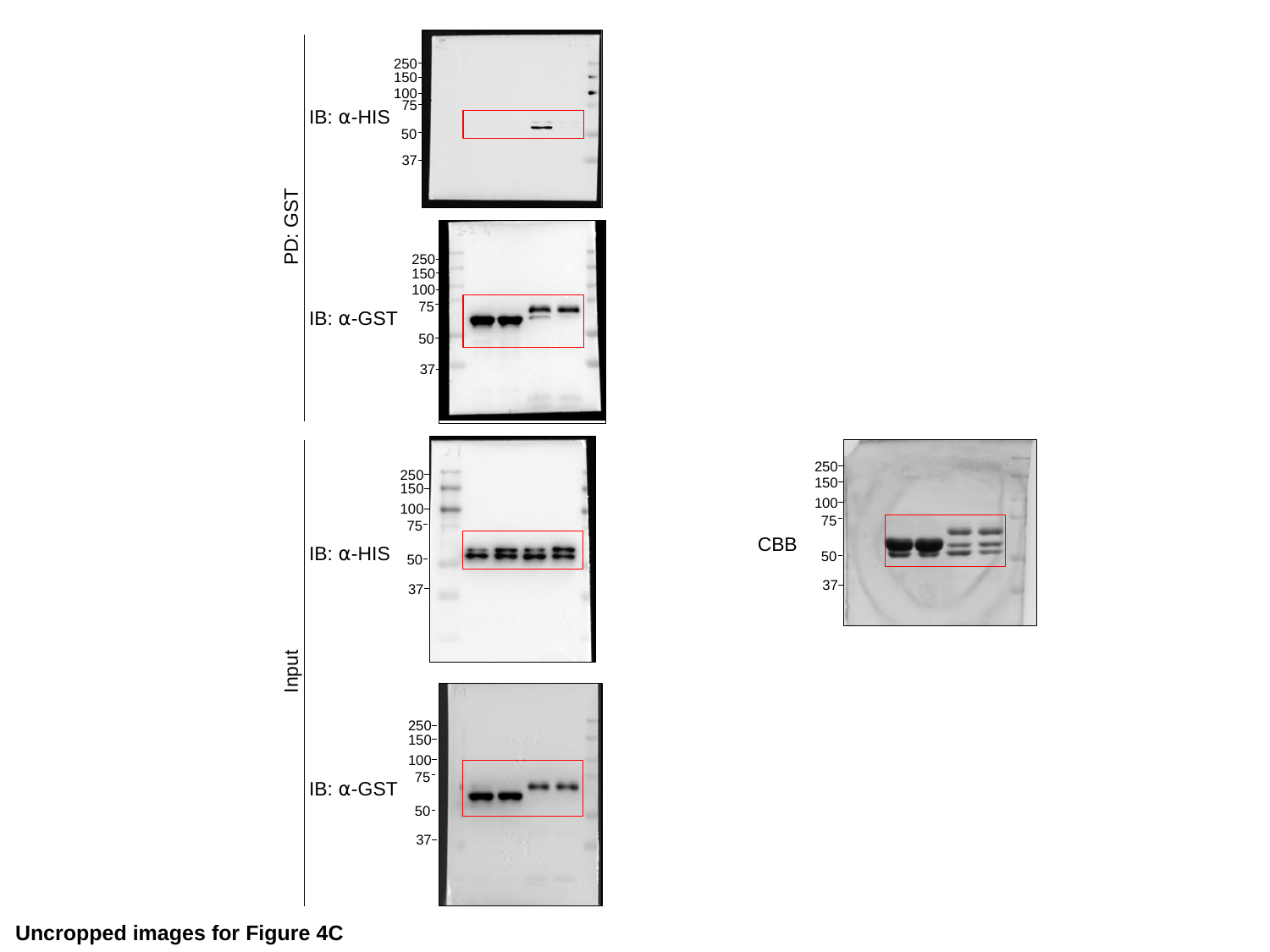

250
150
100
75
IB: ⍺-HIS
50
37
PD: GST
250
150
100
75
IB: ⍺-GST
50
37
Input
250
250
150
150
100
100
75
75
CBB
IB: ⍺-HIS
50
50
37
37
250
150
100
75
IB: ⍺-GST
50
37
Uncropped images for Figure 4C

## Slide 4
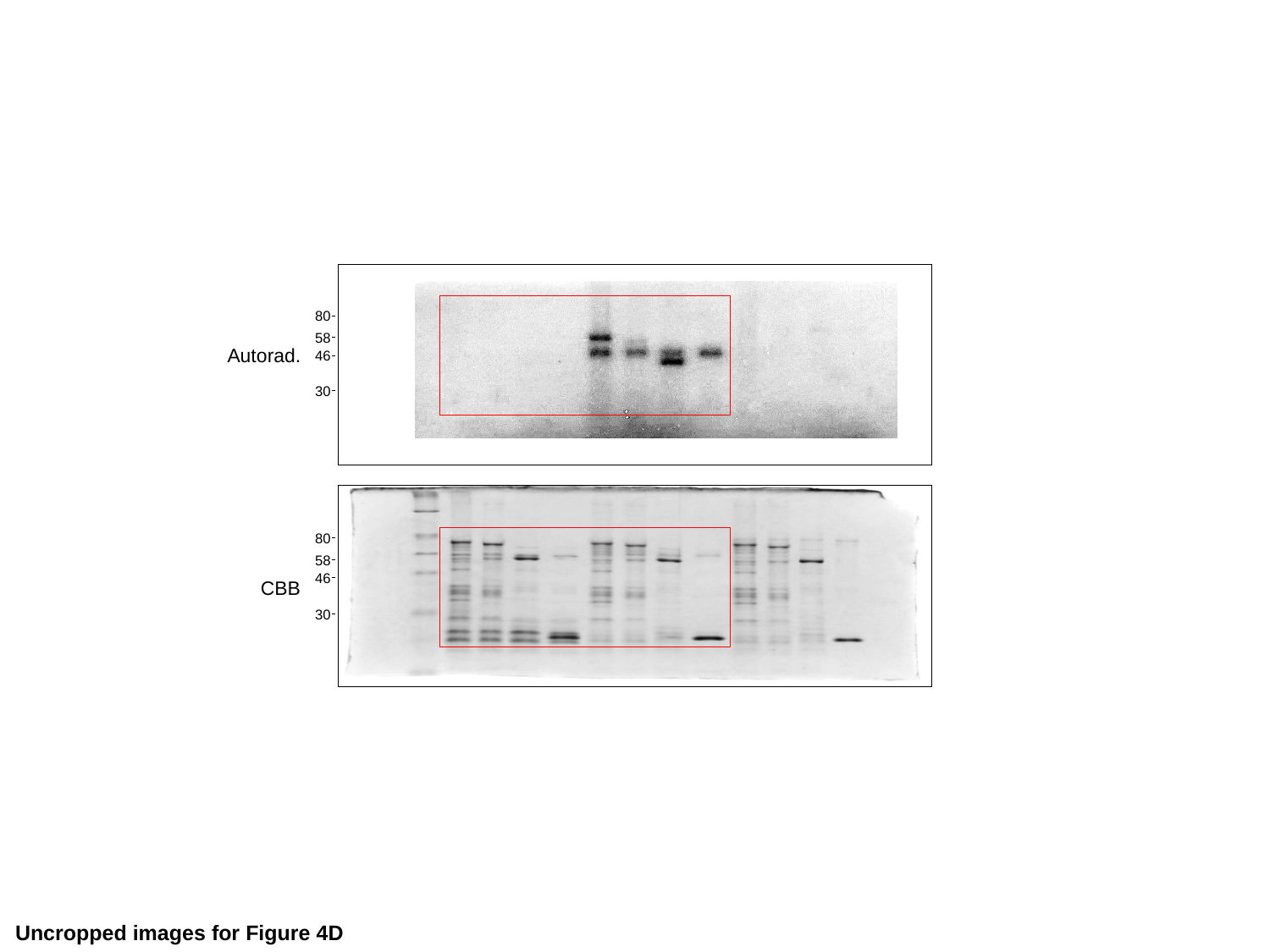

80
58
Autorad.
46
30
80
58
46
CBB
30
Uncropped images for Figure 4D

## Slide 5
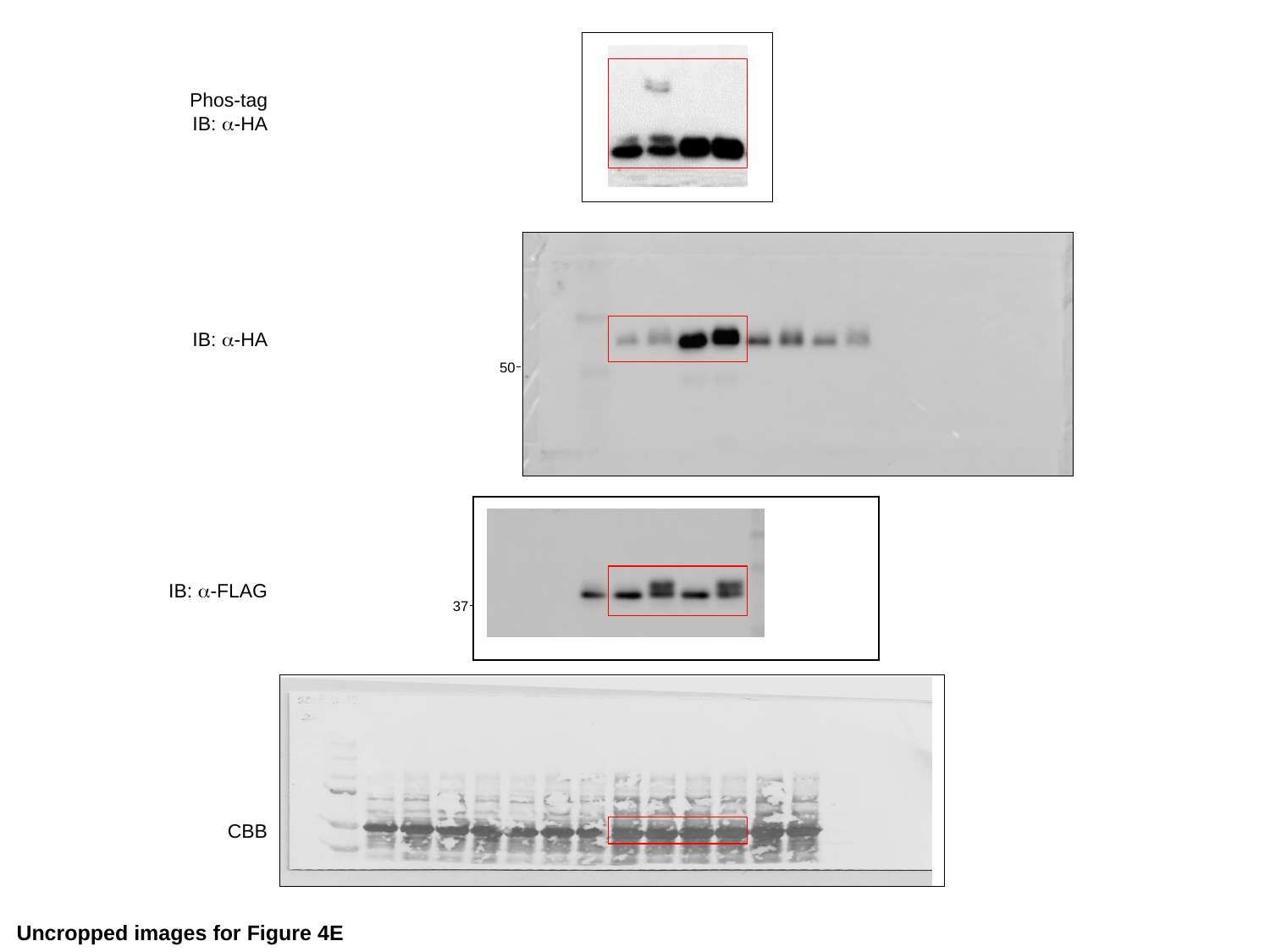

Phos-tag
IB: α-HA
IB: α-HA
50
IB: α-FLAG
37
CBB
Uncropped images for Figure 4E

## Slide 6
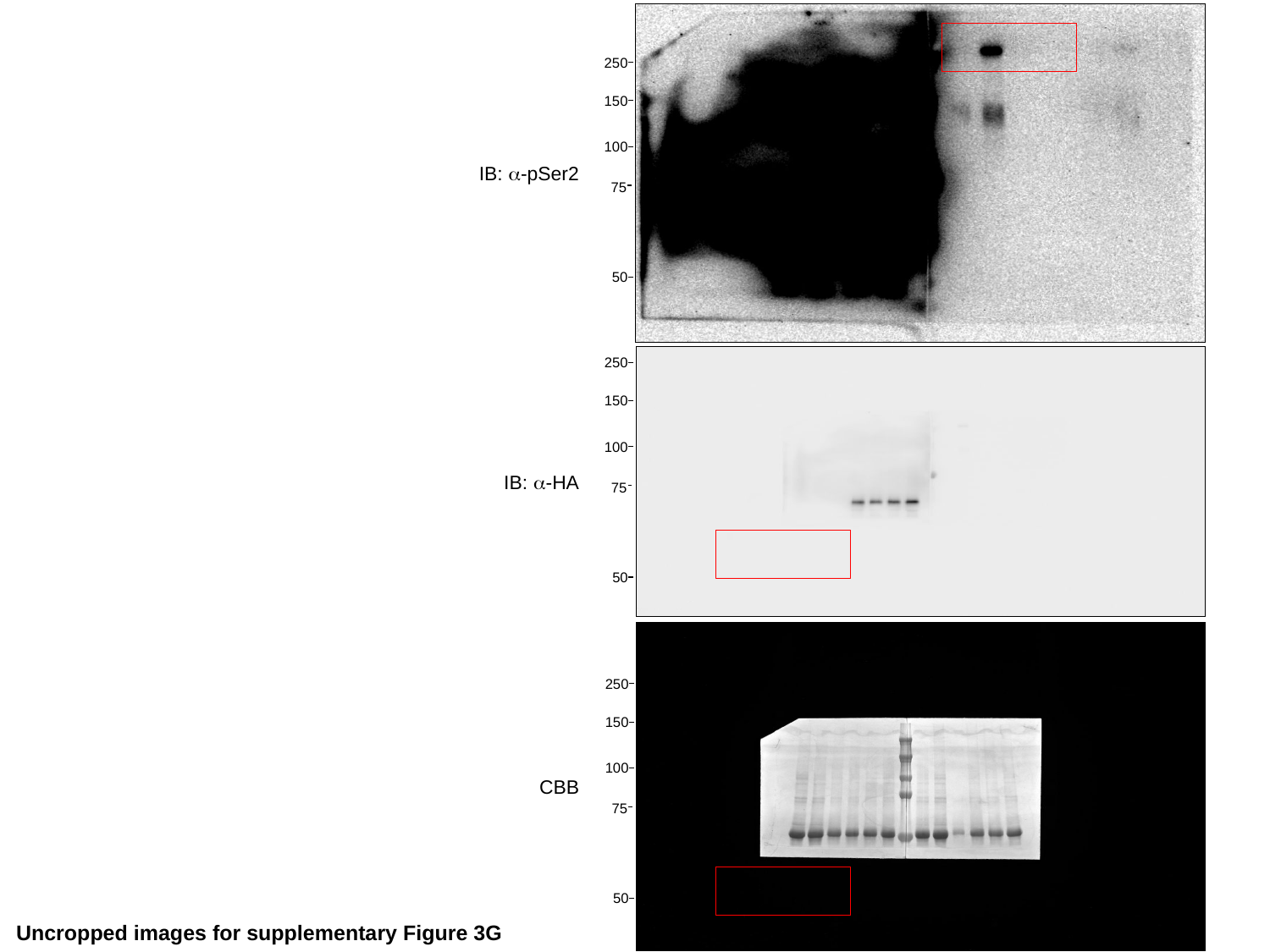

250
150
100
IB: α-pSer2
75
50
250
150
100
IB: α-HA
75
50
250
150
100
CBB
75
50
Uncropped images for supplementary Figure 3G
